# Development of Novel 4-Arylpyridin-2-one and 6-Arylpyrimidin-4-one Positive Allosteric Modulators of the M_1_ Muscarinic Acetylcholine Receptor

**DOI:** 10.1002/cmdc.202000540

**Published:** 2020-09-25

**Authors:** Manuela Jörg, Elham Khajehali, Emma T. van der Westhuizen, K. H. Christopher Choy, David Shackleford, Andrew B. Tobin, Patrick M. Sexton, Celine Valant, Ben Capuano, Arthur Christopoulos, Peter J. Scammells

**Affiliations:** a Medicinal Chemistry, Monash Institute of Pharmaceutical Sciences Monash University, Parkville 3052, Victoria (Australia); b Drug Discovery Biology, Monash Institute of Pharmaceutical Sciences, Monash University, Parkville 3052, Victoria (Australia); c Centre for Drug Candidate Optimisation, Monash Institute of Pharmaceutical Sciences, Monash University, Parkville 3052, Victoria (Australia); d Centre for Translational Pharmacology, Institute of Molecular, Cell and Systems Biology, College of Medical, Veterinary and Life Sciences, University of Glasgow, Glasgow, G12 8QQ (UK)

**Keywords:** allosteric ligands, modulators, muscarinic acetylcholine receptor

## Abstract

This study investigated the structure-activity relationships of 4-phenylpyridin-2-one and 6-phenylpyrimidin-4-one muscarinic M_1_ acetylcholine receptor (M_1_ mAChRs) positive allosteric modulators (PAMs). The presented series focuses on modifications to the core and top motif of the reported leads, MIPS1650 (**1**) and MIPS1780 (**2**). Profiling of our novel analogues showed that these modifications result in more nuanced effects on the allosteric properties compared to our previous compounds with alterations to the biaryl pendant. Further pharmacological characterisation of the selected compounds in radioligand binding, IP_1_ accumulation and β-arrestin 2 recruitment assays demonstrated that, despite primarily acting as affinity modulators, the PAMs displayed different pharmacological properties across the two cellular assays. The novel PAM **7f** is a potential lead candidate for further development of peripherally restricted M_1_ PAMs, due to its lower blood–brain-barrier (BBB) permeability and improved exposure in the periphery compared to lead **2**.

## Introduction

Muscarinic acetylcholine receptors (mAChRs) are G protein-coupled receptors, consisting of five distinct subtypes (M_1_–M_5_).^[1]^ The M_1_, M_3_ and M_5_ mAChRs are preferably coupled to the G_q/11_ proteins that lead to phospholipid hydrolysis to generate the secondary messengers inositol 1,4,5-triphosphate (IP_3_) and diacylglycerol (DAG), whereas the M_2_ and M_4_ mAChRs are preferably coupled to the G_i/o_ proteins that inhibit adenylate cyclase.^[1,2]^ The activation of mAChRs is linked to changes in second-messenger levels and activity of kinases, phospholipases, ion channels, and other membrane receptors.^[3]^

The M_1_ mAChR subtype is expressed in multiple neuronal and non-neuronal cell types located in brain, autonomic ganglia, gastrointestinal tract, secretory glands, vas deferens and the sympathetic ganglia tissue.^[2]^ The activation of M_1_ mAChRs is therefore associated with numerous functions, including seizures, cognition and learning, locomotor activity,^[4]^ apoptotic cell death, and intestinal mobility.^[5]^

Approved pharmaceutical interventions for increasing the levels of the endogenous agonist acetylcholine (ACh) include acetylcholinesterase inhibitors that are used in the treatment of Alzheimer’s disease patients.^[6]^ However, these drugs only provide moderate improvement in cognitive deficits and are associated with numerous adverse effects.

The positive cognitive effects associated with activation of the M_1_ mAChR holds great promise for treatment of diseases such as Alzheimer’s, however, the design of subtype-selective ligands has been extremely challenging, partly due to the highly conserved orthosteric binding site across the five mAChR subtypes.^[7]^ Consequently, the concept of targeting less conserved and topographically distinct allosteric regions of the M_1_ mAChR has gained attention. Allosteric ligands possess a unique pharmacological profile as they can modulate the binding and/or signalling activity of orthosteric agonists, and might also activate the receptor in their own right (i.e., allosteric agonism).^[8]^ Furthermore, targeting allosteric binding sites can yield M_1_ mAChR PAMs with good subtype selectivity, therefore this class of ligand might also cause fewer off target mAChR-dependent side-effects compared to traditional orthosteric agonists.^[8]^

BQCA was the first highly selective M_1_ mAChR PAM reported in the literature and was subsequently used in preclinical proof-of-concept studies, displaying activity in animal models of cognitive deficits.^[9]^ Consequently, extensive research efforts have been made by groups in academia and industry to improve our understanding of the specific mechanisms of action of this compound class, and also to develop new scaffolds with higher affinity and improved physicochemical properties.^[10]^ To date, M_1_ mAChR PAMs have mostly been investigated for the treatment of cognitive deficits in the central nervous system (CNS); disorders such as Alzheimer’s disease and schizophrenia. However, more recently, M_1_ mAChR PAMs have been shown to induce coordinated colonic propulsive activity and defecation and it has been proposed that peripherally restricted M_1_ mAChR PAMs might provide a new therapeutic option for the treatment of constipation.^[11]^

**Figure 1:**
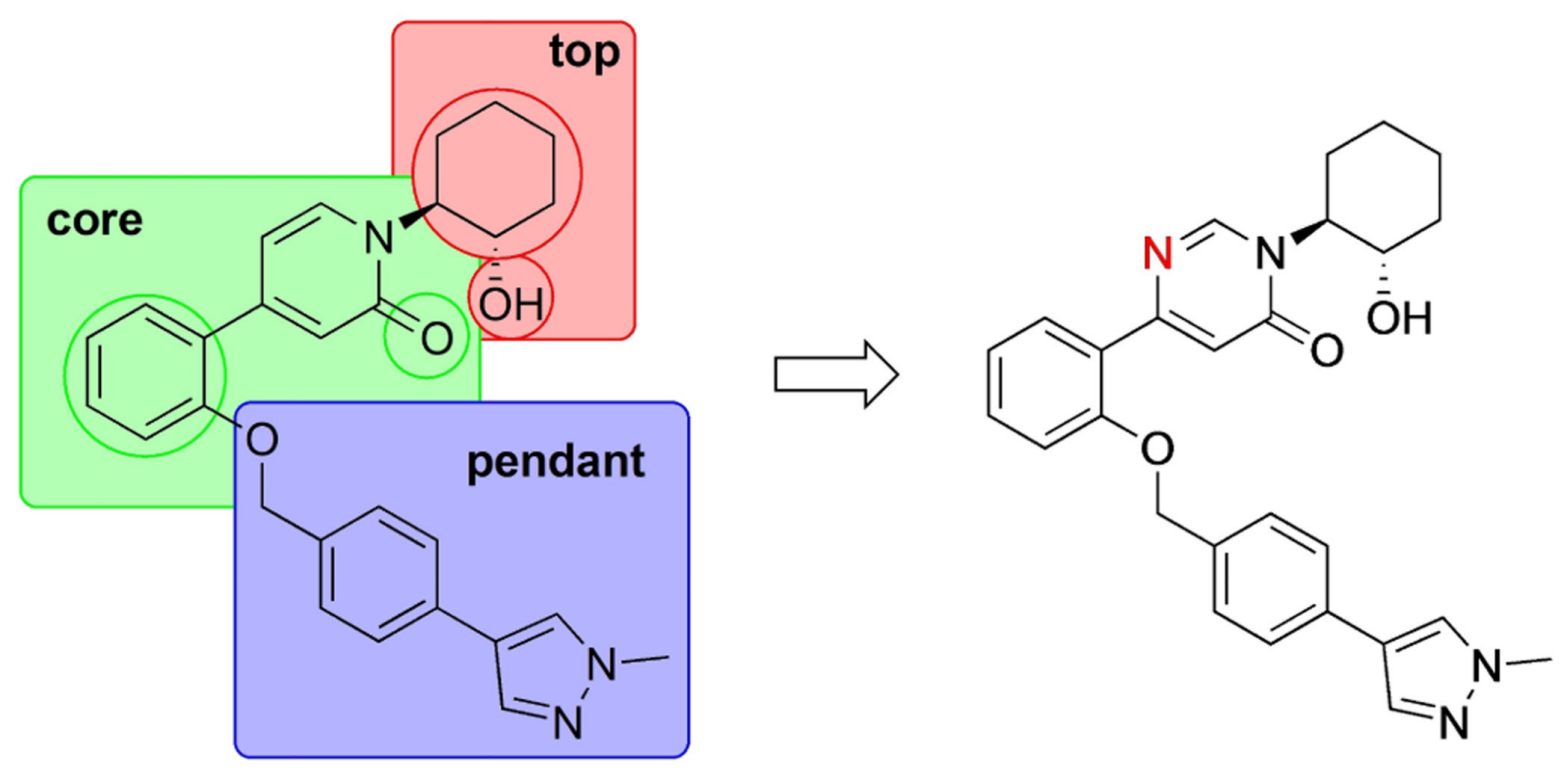
Overview of our approach using the 4-phenylpyridin-2-one, MIPS1650 (**1**) and 6-phenylpyrimidin-4-one, MIPS1780 (**2**) as lead compounds.

Previously, our group reported 4-phenylpyridin-2-ones as novel PAMs at the M_1_ mAChR, with MIPS1650 (**1**) as the lead compound (Figure 1). Subsequently, an extensive series of 4-phenylpyridin-2-one analogues with modifications to the pendant motif were synthesized and their pharmacology was evaluated, revealing compounds with different allosteric properties.^[10a]^ Modification of the heterocyclic core also led to the discovery of the first 6-phenylpyrimidin-4-one analogue, MIPS1780 (**2**), as a novel M_1_ mAChR PAM scaffold.^[10a,12]^ The introduction of the additional nitrogen atom in the core, produced a fourfold increase in binding cooperativity with ACh (*α*_ACh_) and 11-fold increase in intrinsic efficacy (*τ*_B_) compared to MIPS1650 (**1**).^[10a]^

In this study, we have further explored the structure–activity relationships of 4-phenylpyridin-2-one and 6-phenylpyrimidin-4-one PAMs, investigating a range of alterations to their core and top motif (Figure 1). In the first instance, we looked at modifications to core and top part of the 4-phenylpyridin-2-one, lead **1**. Modifications which influenced allosteric effects were subsequently introduced to the 6-phenylpyrimidin-4-one scaffold.

## Results and Discussion

### Chemistry

**Scheme 1:**
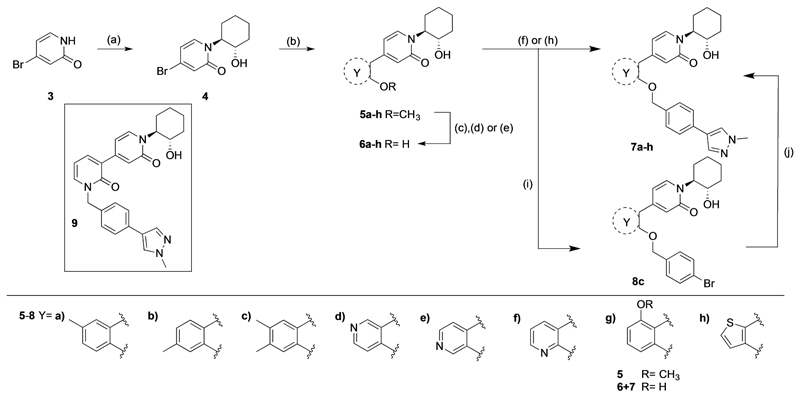
Synthesis of 4-phenylpyridin-2-one analogues with modification to the core motif. a) 1,2-Cyclohexene oxide, K_2_CO_3_, 120°C, 77% **4** (*rac-trans*); b) respective boronic acid or pinacol ester, cat. PdCl_2_(PPh_3_)_2_, 1 M Na_2_CO_3(aq)_/THF degassed, 100°C, 28–90%; c) 1 M BBr_3_ in hexane, CH_2_Cl_2_, 0°C to RT, 39–100%; d) *p*-TsOH, LiCl, NMP, microwave, 180°C, 19% (**6d**) and 11% (**6e**); e) HBr/EtOH 1:1, 70°C, 93% (**6f**); f) 4-(4-(chloromethyl)phenyl)-1-methyl-1*H*-pyrazole, K_2_CO_3_, DMF, RT, 2–48%; g) 4-(4-(chloromethyl)phenyl)-1-methyl-1*H*-pyrazole, Ag_2_CO_3_, DMF, 70°C, 21% (**7f**); h) 4-bromobenzyl bromide, K_2_CO_3_, KI, DMF, RT, 51%; i) 1-methylpyrazole-4-boronic acid pinacol ester, cat. PdCl_2_(PPh_3_)_2_, 1 M Na_2_CO_3(aq)_/THF degassed, 100°C, 48%.

The optimized synthesis of (±)-*trans*-4-bromo-1-(2-hydroxycy-clohexyl)pyridin-2(1*H*)-one (**4**) starting from the commercially available 4-bromopyridin-2(1*H*)-one (**3**) was previously reported by our group (Scheme 1).^[10a]^ The epoxide ring-opening reaction was performed under neat reaction conditions at 120°C using 5–10 equivalents of the 1,2-cyclohexene oxide, yielding 77% of the desired N-linked isomer **4** (only the *trans*-isomers were formed in a racemic mixture). Intermediates **5a**–**h** were formed via Suzuki coupling reactions of **4** and the respective *o*-methoxyarylboronic acids or pinacol esters in yields ranging from 28–90%. A number of different reaction conditions were needed to demethylate intermediates **5a**–**h** to the corresponding phenols **6a**–**h**, as the previously reported method using 1 M boron tribromide in hexane was not effective for the pyridines **5d**–**f**. Methoxypyridines **5d**–**e** were converted to the corresponding phenols **6d**–**e** in low yields (11–19%) using an excess of *p*-toluenesulfonic acid and lithium chloride in NMP at 180°C, whereas **6f** was obtained in 93% yield after treatment with a 1:1 mixture of hydrobromic acid (48% in water) and ethanol.

Lastly, the 4-phenylpyridin-2-one analogues **7a**–**h** were either obtained via direct alkylation with 4-(4-(chloromethyl) phenyl)-1-methyl-1*H*-pyrazole,^[13]^ or a two-step procedure, including the O-alkylation with 4-bromobenzyl bromide to give **8c** followed by a Suzuki coupling reaction with 1-methylpyrazole-4-boronic acid pinacol ester. The alkylation of all final analogues apart from **7f** was performed using potassium carbonate in DMF at room temperature. The hydroxypyridine functionality of intermediate **6f** permits N- and O-alkylation; the use of potassium carbonate predominately formed the N-alkylated analogue **9**, whereas silver carbonate in DMF at 70°C afforded **7f** in 21% yield.

**Scheme 2:**
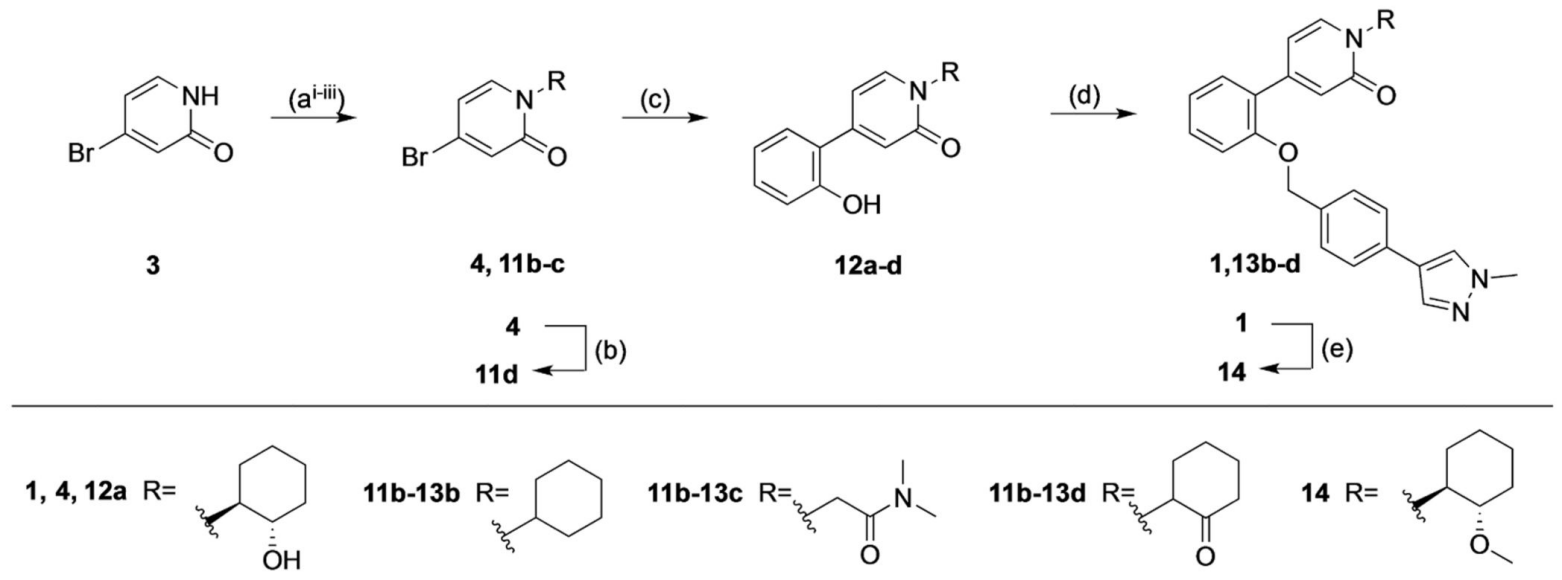
Synthesis of final analogues with modification to the top motif. a^i^) 1,2-Cyclohexene oxide, K_2_CO_3_, 120°C, 77% of **4** (*rac-trans*); a^ii^) bromocyclohexane, K_2_CO_3_, 120°C, 77% (**11b**); a^iii^) 2-bromo-*N,N*-dimethylacetamide, K_2_CO_3_, KI, RT, 97% (**11c**); b) Dess-Martin periodinane, CH_2_Cl_2_, 0°C to RT, 96%; c) (2-hydroxyphenyl)boronic acid, cat. PdCl_2_(PPh_3_)_2_, 1 M Na_2_CO_3(aq)_/THF degassed, 100°C, 14–83%; d) 4-(4-(chloromethyl)phenyl)-1-methyl-1*H*-pyrazole, K_2_CO_3_, DMF, RT, 15–72%; e) MeI, NaH, CH_2_Cl_2_, RT, 25%.

The 4-phenylpyridin-2-one analogues with modifications to the top motif (Scheme 2) were synthesized from 4-bromopyridin-2(1*H*)-one (**3**) either by an epoxide ring-opening reaction to afford cyclohexan-1-ol **4** or an alkylation under alkaline reaction conditions to afford cyclohexane **11b** and *N,N*-dimethylacetamide **11c**. Oxidation of the alcohol **4** with Dess–Martin periodinane afforded the ketone **11d** in excellent yield (96%). Next, intermediates **12a**–**d** were obtained from a Suzuki coupling reaction with (2-hydroxyphenyl)boronic acid in yields ranging from 14–83%. Lead **1** and novel analogues **13b**–**d** were obtained by alkylation with the previously synthesized 4-(4-(chloromethyl)phenyl)-1-methyl-1*H*-pyrazole^[13]^ in moderate to good yields (15–72%). Lastly, alcohol **1** was O-alkylated with methyl iodide, using sodium hydride as the base to afford compound **14** in 25% yield.

**Scheme 3:**
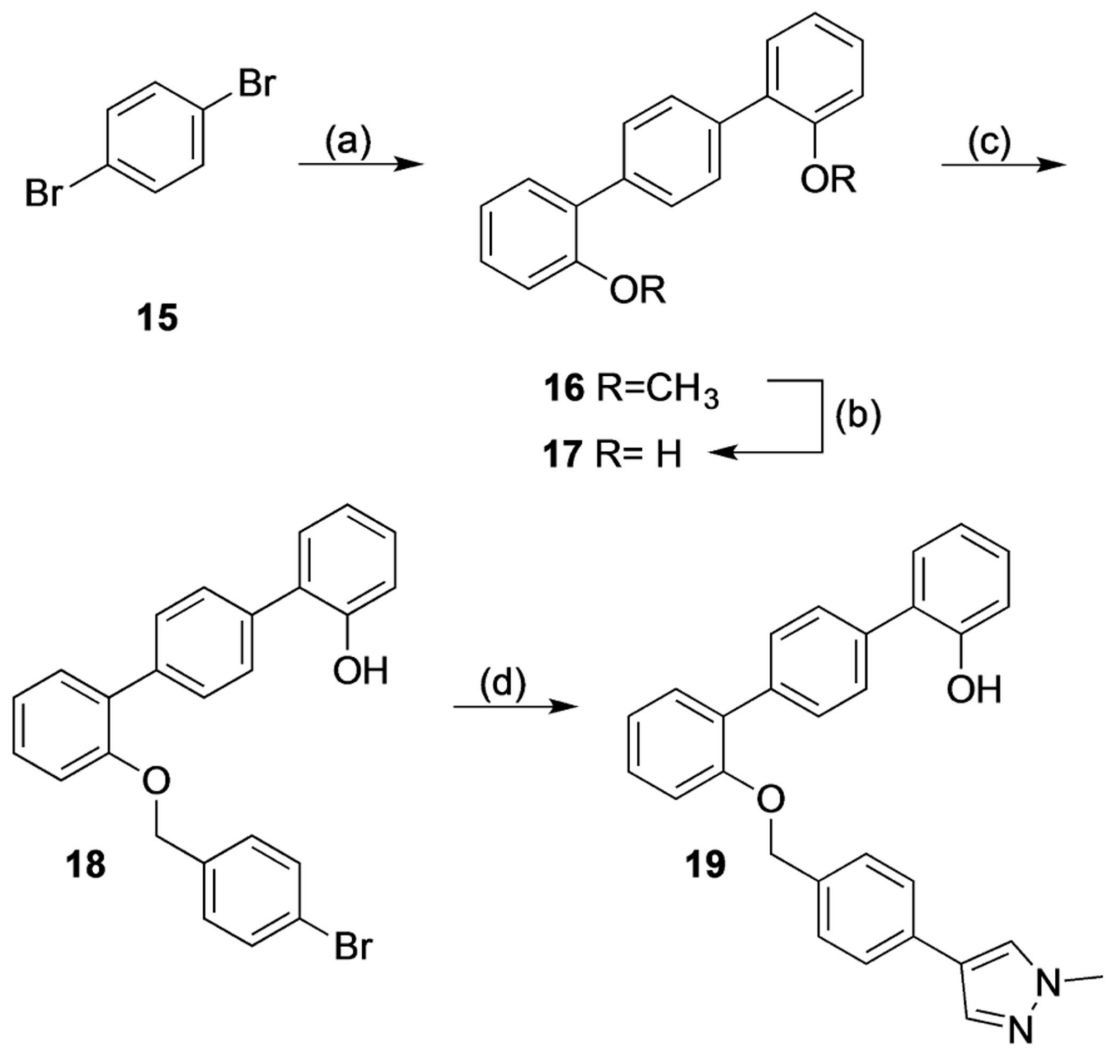
Synthesis of **19**. a) (2-Methoxyphenyl)boronic acid, cat. PdCl_2_(PPh_3_)_2_, 1 M Na_2_CO_3(aq)_/THF degassed, 100°C, 68%; b) 1 M BBr_3_ in hexane, CH_2_Cl_2_, 0°C to RT, 90%; c) 4-bromobenzyl bromide, K_2_CO_3_, KI, DMF, RT, 36%; d) 1-methylpyrazole-4-boronic acid pinacol ester, cat. PdCl_2_(PPh_3_)_2_, 1 M Na_2_CO_3(aq)_/THF degassed, 100°C, 25%.

For the synthesis of analogue **19** (Scheme 3), standard Suzuki coupling reaction conditions were used to convert the commercially available 1,4-dibromobenzene (**15**) to 2,2″-dimethoxy-1,1′:4′,1″-terphenyl (**16**) in 68% yield. The methoxy groups of **16** were demethylated using boron tribromide to obtain **17** in good yield (90%). Next, 4-bromobenzyl bromide was added portion-wise to a solution of **17**, potassium carbonate and potassium iodide in DMF to afford intermediate **18**, with minimal formation of the bis-alkylated side product. Lastly, a Suzuki coupling reaction with 1-methylpyrazole-4-boronic acid pinacol ester was performed to obtain analogue **19** in moderate yield (25%).

**Scheme 4:**
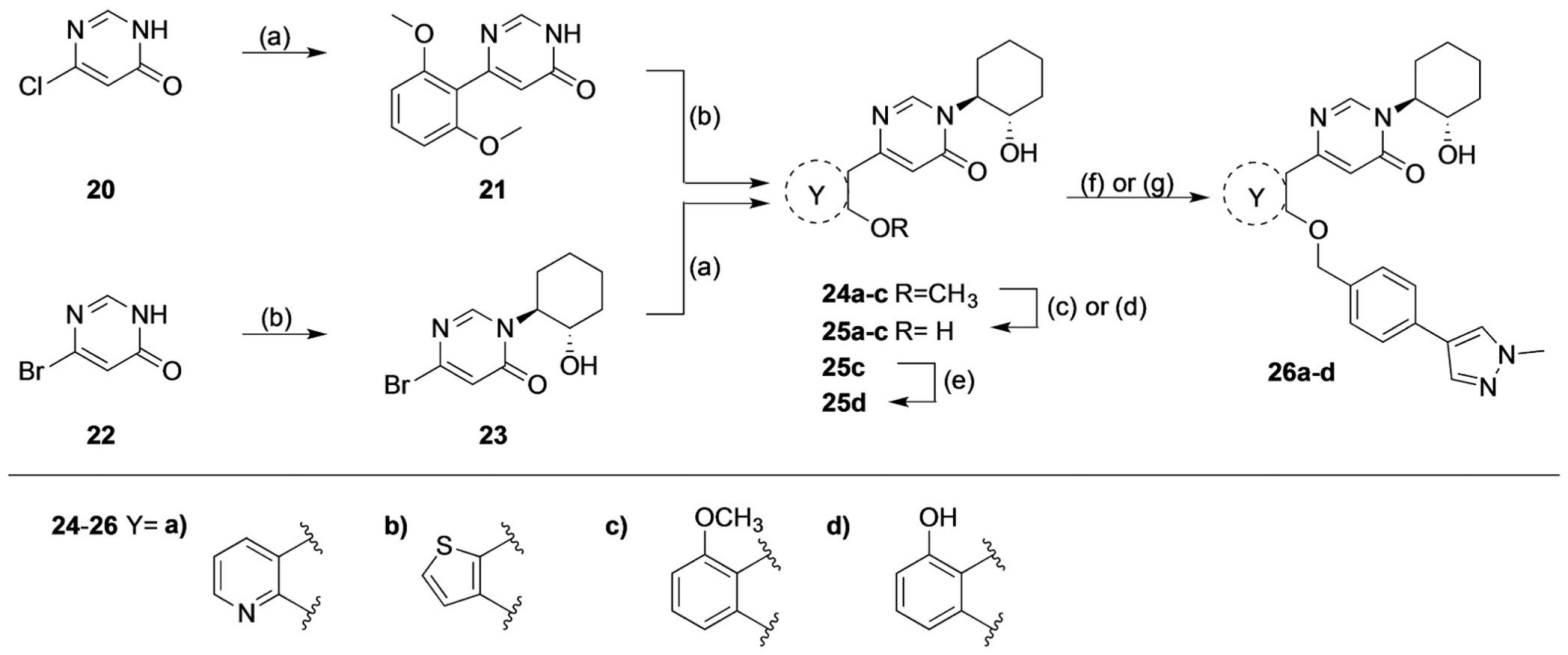
Synthesis of 6-phenylpyrimidin-4-one analogues with modification to the core part. a) Respective boronic acid or pinacol ester, cat. PdCl_2_(PPh_3_)_2_, 1 M Na_2_CO_3(aq)_/THF degassed, 100°C, 18–46%; b) 1,2-cyclohexene oxide, K_2_CO_3_, 120°C, 32% (**21**) and 51% (**25c**); c) HBr/EtOH 1:1, 70°C, 100% (**25a**); d) 1 M BBr_3_ in hexane, CH_2_Cl_2_, 0°C to RT, 73% (**25b**) and 87% (**25c**); e) *p*-TsOH, LiCl, NMP, microwave, 180°C, 34% (**25d**); f) 4-(4-(chloromethyl)phenyl)-1-methyl-1*H*-pyrazole, Ag_2_CO_3_, DMF, 70°C, 7% (**26a**); g) 4-(4-(chloromethyl)phenyl)-1-methyl-1*H*-pyrazole, K_2_CO_3_, DMF, RT, 34–47%.The methoxythiophene **24a** was demethylated with HBr to afford **25a**, while boron tribromide was used to demethylate **24b** and **24c** to afford **25b** and **25c**, respectively. Compound **24d** was resistant to demethylation of both methoxy groups by these methods, but ultimately treatment of **25c** with *p*-toluenesulfonic acid and lithium chloride in NMP at 180°C, yielded **25d** in a moderate 34% yield. Finally, intermediates **25a**–**d** were alkylated with 4-(4-(chloromethyl)phenyl)-1-methyl-1*H*-pyrazole^[13]^ using either silver carbonate or potassium carbonate as the base to obtain analogues **26a** and **26b**–**d**, respectively.

The synthesis of pyrimidinone **23** from the commercially available 6-bromopyrimidin-4(3*H*)-one (**22**) has previously been reported by our group (Scheme 4).^[14]^ It was shown that the more nucleophilic 6-bromopyrimidin-4-one **22** (compared to **20**) was essential to promote an epoxide ring opening reaction to form both the N-alkylated and O-alkylated products in a *trans* isomer racemic mixture in a 2:1 ratio.^[14]^ The advantage of this synthetic route is the initial instalment of the 1,2-cyclohexene oxide functionality, followed by the Suzuki reaction coupling reaction with the more expensive *ortho*-methoxyaromatic moieties to afford intermediate **24a**–**b**. The reverse synthetic pathway–Suzuki coupling reaction, followed by epoxy ring-opening reaction with 1,2-cyclohexene oxide - was used to obtain intermediate **24c** as the (2,6-dimethoxyphenyl)boronic acid was readily available. Next, demethylation of meth-oxypyridine **24a** was achieved in a 1:1 mixture of hydrobromic acid (48% in water) and ethanol at 70°C resulting in intermediate **25a**.

**Scheme 5:**
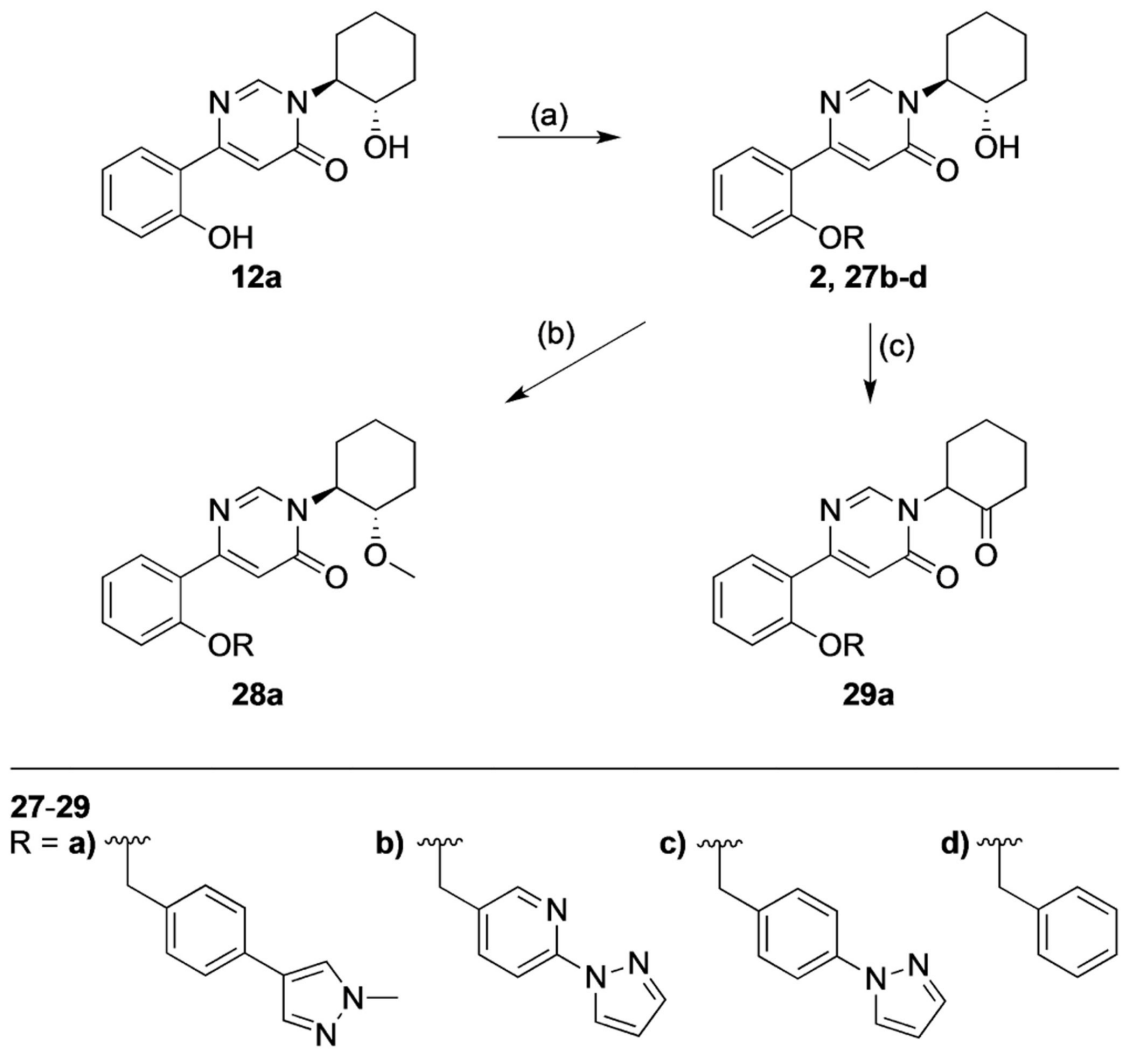
Synthesis of 6-phenylpyrimidin-4-one analogues with modification to the top and pendant moiety. a) benzyl halide, K_2_CO_3_, DMF, RT, 71–89%; b) MeI, NaH, CH_2_Cl_2_, RT, 45%; c) Dess–Martin periodinane, CH_2_Cl_2_, 0°C to RT, 41%.

Lastly, a series of pyrimidin-4-one analogues were synthe-sized with three different pedant motifs, and modifications to the hydroxy group of the cyclohexanol moiety of **27b** were also prepared (Scheme 5). Therefore, the previously synthesized intermediate **12a**^[10a,14]^ was alkylated with the respective benzyl halide in DMF at room temperature to obtain **2** and **27b**–**d**. The hydroxy group was either methylated with iodomethane to afford **28a** in moderate yield (45%) or converted to the corresponding ketone via oxidation with Dess–Martin periodinane to obtain **29a** in 41% yield.

### Primary pharmacological screening

Functional activity of the synthesized analogues as racemic mixtures was investigated in IP_1_ accumulation assays at the M_1_ mAChR, as described previously.^[13–14]^ ACh concentration-response curves were generated in the absence or presence of a 1 and 10 μM concentration of each modulator. The change in baseline activity (Δbaseline) is an approximate measure of direct allosteric agonism (*τ*_B_), whereas the pEC_50_ shift (ΔpEC_50_) is an approximate measure of functional cooperativity (*αβ*) of each modulator with ACh in the investigated signalling pathway. We aimed to identify modulators with various degrees of allosteric agonism and modulatory effects on ACh response, as these could generate distinct *in vivo* outcomes for applications in different conditions in the CNS or gastrointestinal (GI) tract.^[10g,h,j]^

Table 1 shows the effect of modifications to the core of MIPS1650 (**1**). The addition of a methyl substituent in one or both, 4- and 5-position as depicted in compounds **7a, 7b, 7c**, drastically reduced their allosteric agonism and modulatory effects on ACh-induced IP_1_ accumulation. A range of effects were observed for the pyridine compounds **7d, 7e** and **7f** depending upon the position of the nitrogen in the ring. The addition of a nitrogen in the *para*-position to the substituted benzyloxy functionality, as in **7d**, resulted in a loss of function compared with **1**, whereas moving the nitrogen to the *meta* position (**7e**) had a substantially differential effect, increasing the allosteric agonism of the compound. On the other hand, the 2-alkoxypyridine motif in **7f** reduced the intrinsic agonism of the allosteric modulator, while maintaining the allosteric potentiation of the ACh response. The drastic changes in the pharmacological profile of these compounds, by moving the nitrogen by one position, suggests that the pyridine nitrogen is involved in an important interaction with the M_1_ mAChR allosteric site. The introduction of a hydroxy group *ortho* to the 1-(2-hydroxycyclohexyl)pyridin-2(1*H*)-one group (compound **7g**) caused a significant drop in the compound’s ability to modulate ACh response compared to the parent compound **1**, suggesting that substituents in this position might not be well tolerated. In contrast, changing the 1,2-phenylene to a 2,3-disubstituted thiophene as depicted in **7h**, increased the allosteric agonism and potentiation of the ACh response compared to parent compound **1** at the concentrations investigated. Last, the structurally altered N-alkylated analogue **9** exhibited reduced positive allosteric modulation compared to its O-alkylated counterpart **7f**. From this series of compounds, **7f** and **7h** were selected for further pharmacological testing.

The effect of modifications of this type to the 6-arylprimi-din-4-one core of MIPS1780 (**2**) were also investigated and the findings are summarised in Table 2. In this case changing the 1,2-phenylene unit present in **2** to a 2,3-disubstituted pyridine (**26a**) or thiophene (**26b**) resulted in high agonist activity which reached 100% ACh maximal response (*E*_max_). In contrast, the methoxy analogue **26c** exhibited minimal potentiation of the ACh response or allosteric agonism. The phenol **26d** potentiated the ACh response to a similar extent to the parent **2** and has a more pronounced effect on the baseline response.

Table 3 shows the results of changes to the cyclohexanol moiety of the parent compound **1**. Removal of the hydroxy group from the cyclohexyl ring (compound **13b**) caused a complete loss of allosteric function. Replacing the top motif with a *N,N*-dimethylacetamide as in **13c**, preserves the activity of the modulator, resulting in an allosteric profile comparable to lead **1**. Replacing the secondary alcohol with a ketone (compound **13d**) reduced allosteric agonism at 1 and 10 μM, while maintaining potentiation of the ACh response at 10 μM. The methoxy analogue **14** exhibited some capacity to potentiate ACh and act as an allosteric agonist, but its activity was reduced relative to **1**. Lastly, the terphenyl analogue **19** exhibited little or no activity. The incorporation of a polar moiety in the top motif seems to be important for activity of this class of M_1_ mAChR allosteric modulators, based on the observation that replacing the hydroxy group with ketone (**13d**) or methoxy (**14**) functionality was tolerated to varying degrees, while the cyclohexane analogue (**13b**) exhibited a total loss of activity. This would also explain the observed results for *N,N*-dimethylacetamide analogue **13c**, which also contains a carbonyl group. In summary, **13 c, 13 d** and **14** were the only compounds from our series with modifications to the top motif of the parent compound **1**, which displayed comparable or reduced allosteric agonism and maintained allosteric potentiation of the ACh-stimulated IP_1_ response at 1 and/or 10 μM.

As some alterations to the hydroxy group, specifically to a oxo or methoxy group, on our 4-phenylpyridin-2-one lead **1** maintained the allosteric potentiation, but significantly reduced allosteric agonism, the same modifications were investigated on the 6-phenylpyrimidin-4-one lead **2** (Table 3). Modification to the hydroxy group in lead **2** produced different effects compared to lead **1**. More specifically, both the ketone analogue **28a** and the methoxy analogue **29a** exhibited drastically reduced allosteric potentiation, but retained good allosteric agonism.

Finally, a number of biaryl pendants were also explored (Table 4), including the 4-(1-methylpyrazole-4-yl)benzyl (**2, 28 a** and **29a**), the structurally related 4-(1*H*-pyrazole)pyridylmethyl (**27b**) and 4-(1*H*-pyrazole)benzyl (**27c**) as well as a benzyl pendant (**27d**). The latter was introduced based on our previous findings that **27d** has similar capacity to **2** for modulation of ACh response, while exhibiting less allosteric agonism.^[14]^ Compound **27b** exhibited a similar profile to the parent compound **2** at 10 μM, whereas compound **27d** showed a similar capacity to modulate the Ach response, but significantly reduced allosteric agonism. Allosteric metrics were not determined for **27c** due to due to high agonist activity of the modulator, reaching 100% of the ACh maximal response.

### Characterization of selected analogues in radioligand binding, IP_1_ accumulation and β-arrestin 2 recruitment assays

We selected six compounds (**7f, 7h, 13c, 13d, 26a** and **27a**) that displayed varying degrees of allosteric agonism in the primary pharmacological screen for further pharmacological evaluation. This test set of new M_1_ PAMs was comprised of four 4-phenylpyridin-2-ones with modifications to the core (**7f** and **7h**) and top (**13c** and **13d**) as well as two 6-phenylpyrimidin-4-ones with modifications to the core (**26a**) and pendant (**27b**). These compounds underwent detailed analysis in parallel with compounds **1** and **2** and the reference M_1_-selective PAM, BQCA in radioligand binding assays and two functional assays (IP_1_ accumulation and β-arrestin 2 recruitment).

**Figure 2:**
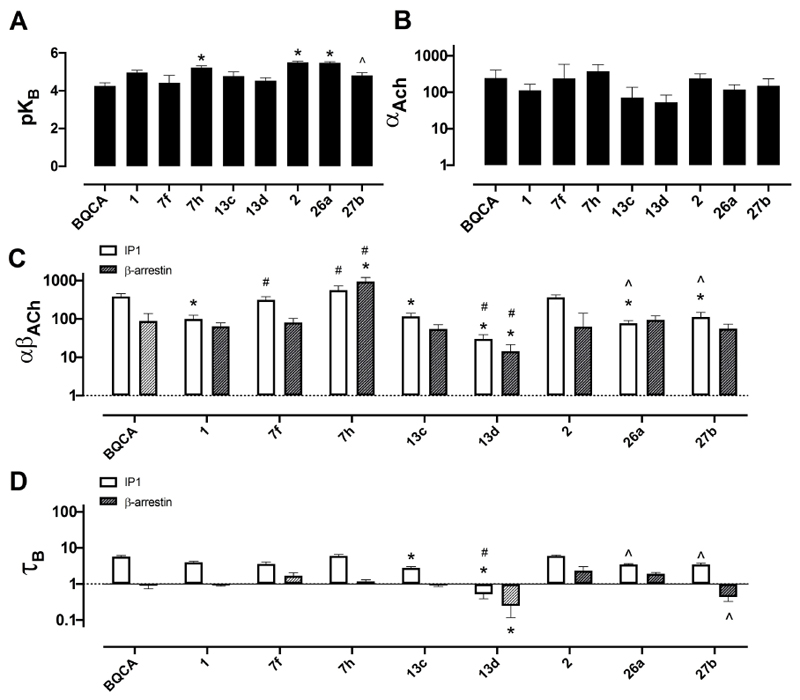
Binding and functional parameter estimates for selected PAMs at the M_1_ mAChR. A) Binding affinity values expressed as negative logarithm. B) Binding cooperativity between ACh and each modulator. C) Functional cooperativity between ACh and each modulator in IP_1_ and β-arrestin 2 assays. D) Intrinsic efficacy of the modulator in IP_1_ and β-arrestin 2 assays. Data represent the mean+SEM of at least three individual experiments performed in duplicate. * Significantly different from the corresponding values for BQCA as the reference PAM, one-way ANOVA with Dunnett’s posthoc test. ^#^ Significantly different from the corresponding values for lead compound **1**, one-way ANOVA with Dunnett’s post-hoc test. ■■Significantly different from the corresponding values for lead compound **2**, one-way ANOVA with Dunnett’s post-hoc test■ ■

To determine the affinity (p*K*_B_) of the allosteric modulators for the allosteric site, and their binding cooperativity with ACh (log *α*_*ACh*_) at the M_1_ mAChR, whole cell equilibrium competition binding studies were performed, using [^3^H]NMS to label the orthosteric site. The data were analysed using an allosteric ternary complex model [Eq. (2) in the Experimental Section, Figure S1 in the Supporting Information],^[15]^ and the estimated values are shown in Table 5 as well as Figure 2A and B. Compounds **2, 7h** and **26a** exhibited higher affinity for the M_1_ mAChR relative to BQCA (Figure 2A). Despite differences in binding affinity values, all the modulators, lead compounds and analogues, globally retained similar binding cooperativity with ACh (Figure 2B).

The compounds were then fully characterised in two functional assays (Figure 2C and D); the G_q_-coupled IP_1_ accumulation assay (Figure S2), which was used for the initial screening of the compounds, and a β-arrestin 2 recruitment assay (Figure S3). The operational efficacy (*τ*_B_) values for the modulators and their functional cooperativity estimates with ACh (log*αβ*_*ACh*_) at both signalling pathways were estimated by applying an operational model of allosterism and agonism to the data [Eq. (3)],^[16]^ and are listed in Table 5. As shown in Figure 2C, varying degrees of functional cooperativity between ACh and modulators were observed in both IP_1_ and β-arrestin 2 assays, with generally lower cooperativity in the arrestin-recruitment assay. BQCA and the lead compound **2** modulate both IP1 accumulation and β-arrestin recruitment to a similar extent. Of note, lead compound **1** displayed significantly lower cooperativity with ACh in IP1 accumulation assay compared to BQCA, but retained cooperativity in β-arrestin 2 recruitment. Thiophene **7h**, showed the highest functional cooperativity with ACh in β-arrestin 2 recruitment, while **13d** exhibited the lowest functional cooperativity for both IP_1_ and β-arrestin 2. Interestingly, **7h** showed increased, and **13d** decreased functional cooperativity estimates compared to the parent molecule **1**. This suggests that changes to the core of compound **1** are able to improve the allosteric modulatory effects of 4-phenylpyridin-2-one analogues, however, changes to the top part of the molecule are less favourable for improving functional cooperativity. Notably, **7f** appeared to display similar cooperativity parameters to BQCA and lead **2**, whilst **13c, 26a** and **27b**, displayed identical cooperativity estimates to lead **1**. Finally, **27b**, which showed reduced properties (Δbaseline and ΔpEC_50_ at 1 μM) in the primary screen, confirmed its lower PAM activity in IP_1_ accumulation compared to lead **2**. Finally, **27b**, which showed reduced properties (Δbaseline and ΔpEC_50_ at 1uM) in the primary screen, confirmed its lower PAM activity in IP1 accumulation compared to lead **2**.

Figure 2D illustrates the different intrinsic efficacy profiles (*τ*_B_) of the modulators in IP_1_ and β-arrestin 2 assays. BQCA, lead compounds **1** and **2**, and analogues **7f**, and **7h** display similar agonist profiles. Compounds **13c** and **13d** have significantly less intrinsic efficacy in IP_1_, with **13d** being also lesser of an agonist in β-arrestin 2, compared to BQCA. Finally, compounds **26a** and **27b** display reduced efficacy in IP_1_ but only compared to lead compound **2**, not BQCA. Interestingly, analogue **13d**, which is the weakest modulator of ACh, is also the only analogue with the lowest efficacy (log *τ*_B_ < 0; *τ*_B_ < 1) in both the functional assays.

Comparing Figure 2C and D, in IP_1_ accumulation assays all of the compounds except **13d** behaved as PAM-agonists, with significant intrinsic efficacy, however, they displayed notably lower minimal to no agonist activity in β-arrestin 2 recruitment assay despite maintaining the potentiation of ACh response. Assessing the degree of functional efficacy (*β*) driving the global *αβ*_ACh_ parameters performing two-tailed *t*-tests between *α*_ACh_ and *αβ*_ACh_ (IP_1_) or *αβ*_ACh_ (β-arr), we observed that that the modulatory effects of the PAMs in functional assays are mainly derived from the modulation of ACh affinity as indicated by lack of significant differences between the binding cooperativity (log*α*) of each modulator and its functional cooperativity (log*αβ*) in IP_1_ or β-arrestin 2 assays (Table 5). Accepting that an IP_1_ accumulation assay is largely more amplified than a β-arrestin 2 recruitment assay, it is therefore not surprising that modulatory effects (*αβ*) were maintained between the two functional assays, but the degree of intrinsic agonism (*τ*_B_) would appear reduced in the low amplified signalling pathway.

### CNS/plasma exposure *in vivo*

We selected lead **2, 7f, 13c** for initial assessment of *in vivo* exposure (Table 6) to determine their suitability for further testing in animal studies for central or peripheral indications requiring selective M_1_ mAChR targeting. Relative to BQCA, our novel M_1_ PAMs **2, 7f** and **13c** exhibited significantly lower concentrations in the brain and brain-to-plasma partitioning ratios (*K*_p_ and *K*_p,uu_, Table 6).

While this limited distribution into the CNS essentially precludes the potential for effective engagement of central M_1_ mAChR, unbound plasma concentrations of **2** were within a similar range to the concentrations required to achieve *in vitro* potency. The same would be expected for **7f** and **13c** (assuming they have similar plasma protein binding), hence these compounds could represent attractive tools to explore peripheral applications of M_1_ mAChR PAMs, for example, in GI disorders.^[11a]^

## Conclusion

We have generated a detailed structure–activity relationship study of novel M_1_ mAChR PAMs by investigating a range of modifications to the top and core motif of the 4-phenylpyridin-2-one as well as the 6-phenylpyrimidin-4-one positive allosteric modulators, previously reported by our group. Unlike our previous structure–activity relationship studies exploring modifications to the 4-(1-methylpyrazol-4-yl)benzyl pendant,^[10a,14]^ which on no occasion resulted in a detrimental loss in allosteric agonists activity, changes to the top and core motif were much more variable. Additionally, modifications were not always transferable across the two investigated scaffolds. In particular, modifications to the hydroxy functionality of the 4-phenyl-pyridin-2-one lead **1** to the ketone **13d** and methoxy analogue **14** maintained the allosteric modulatory effects at 10 μM, whereas the same modification on the 6-phenylpyrimidin-4-one analogues had detrimental effects.

Further characterization in radioligand binding, IP_1_ and β-arrestin 2 recruitment assays for compounds **7f, 7h, 13c, 13d, 26a** and **27b**, which exhibited a range of allosteric profiles in the initial screening assays, indicated that these PAMs mainly modulate ACh binding rather than function. Therefore, despite showing very weak agonist activity in β-arrestin 2 recruitment assays, their functional cooperativity values were generally comparable across IP_1_ accumulation and β-arrestin 2 recruitment assays. Nonetheless, the different pharmacological properties of the PAMs across the two pathways highlights the importance of pathway-dependent effects in screening new allosteric modulators.

Preliminary *in vivo* exposure assessment of the new 4-arylpyridin-2-one as well as the 6-arylpyrimidin-4-one PAMs showed limited blood–brain-barrier permeability but reasonable unbound plasma exposure, which makes these ligands good candidates for further studies on peripheral applications of M_1_ mAChR. In particular, compound **7f**, offers a promising starting point for *in vitro* and *in vivo* studies targeting new therapeutic options for the treatment of constipation disorders.

### Experimental Section

#### Chemistry

Chemicals and solvents were purchased from standard suppliers and used without further purification. Davisil^®^ silica gel (40–63 μm) for flash column chromatography was supplied by Grace Davison Discovery Sciences (Victoria, Australia) and deuterated solvents were purchased from Cambridge Isotope Laboratories, Inc. (USA, distributed by Novachem Pty Ltd, Victoria, Australia).

Reactions were monitored by thin layer chromatography on commercially available precoated aluminium-backed plates (Merck Kieselgel 60 F_254_). Visualisation was by examination under UV light (254 and 366 nm). Organic solvents were evaporated *in vacuo* at ≤ 40°C (water bath temperature).

^1^H NMR spectra were recorded on a Bruker Avance Nanobay III 400 MHz Ultrashield Plus spectrometer at 400.13 MHz. Chemical shifts (δ) are recorded in parts per million (ppm) with reference to the chemical shift of the deuterated solvent. Coupling constants (*J*) are recorded in Hz and the significant multiplicities described by singlet (s), broad singlet (br s), doublet (d), triplet (t), quadruplet (q), broad (br), multiplet (m), doublet of doublets (dd), doublet of triplets (dt) and doublet of doublet of doublets (ddd).

LCMS were run to verify reaction outcome and purity using an Agilent 6120 Series Single Quad coupled to an Agilent 1260 Series HPLC. The following buffers were used; buffer A: 0.1% formic acid in H_2_O; buffer B: 0.1% formic acid in MeCN. The following gradient was used with a Poroshell 120 EC-C_18_ 50×3.0 mm 2.7 micron column, and a flow rate of 0.5 mL/min and total run time of 5 min; 0–1 min 95% buffer A and 5% buffer B, from 1–2.5 min up to 0% buffer A and 100% buffer B, held at this composition until 3.8 min, 3.8–4 min 95% buffer A and 5% buffer B, held until 5 min at this composition. Mass spectra were acquired in positive and negative ion mode with a scan range of 100–1000 *m/z*. UV detection was carried out at 214 and 254 nm. All retention times (*t*_R_) are quoted in minutes. Preparative HPLC was performed using an Agilent 1260 infinity coupled with a binary preparative pump and Agilent 1260 FC-PS fraction collector, using Agilent OpenLAB CDS software (Rev C.01.04), and an Agilent 7 μM XDB-C8 21.2×250 mm column. The following buffers were used unless stated otherwise: buffer A was H_2_O; buffer B was MeCN, with sample being run at a gradient of 5% or 30% buffer B to 100% buffer B over 10 min, at a flow rate of 20 mL/min. All screening compounds were of > 95% purity unless stated otherwise.

#### General procedure A: Suzuki reaction

A mixture of respective aryl halide (1.0 equiv.) and appropriate boronic acid or pinacol ester (1.5 equiv.) in degassed THF/1 M Na_2_CO_3(aq)_ (3 mL/100 mg) was flushed with nitrogen. PdCl_2_(PPh_3_)_2_ (0.1 equiv.) was added and the reaction mixture heating at reflux until full conversion of the starting material was observed by LC-MS. The THF was evaporated under reduced pressure. The residue was dissolved in EtOAc and washed with water (2 × 50 mL) and brine (50 mL).

#### General procedures B: *O*-Alkylation with benzyl halide

The respective phenol (1.0 equiv.), K_2_CO_3_ (1.1 equiv.), (KI (0.1 equiv.) optional) and the appropriately substituted benzyl halide (1.1 equiv.) were stirred in DMF (3 mL/100 mg) at room temperature until the reaction appeared complete (reaction progress was monitored by LC-MS analysis). The reaction mixture was diluted with EtOAc and washed with water (2 × 50 mL) and brine (3 × 50 mL). The organic layer was dried with Na_2_SO_4_, filtered and concentrated under reduced pressure.

#### **General procedures C: Demethylation with BBr**_**3**_

A solution boron tribromide in CH_2_Cl_2_ (1 M, 2.00 equiv.) was added over 10 min at 0°C to a solution of the respective methoxybenzene starting material (1.00 equiv.) in dichloromethane (3 mL/100 mg). The mixture was allowed to warm up to room temperature and was stirred until the reaction appeared complete (reaction progress was monitored by LC-MS analysis) before it was poured onto ice-water. The pH of the solution was adjusted to pH 6 by addition of sat. NaHCO_3_. Dichloromethane (100 mL) was added and the layers were separated. The organic layer was washed with water (2×50 mL) and brine (50 mL) and the solvent was evaporated under reduced pressure (the organic layer was not washed with Na_2_SO_4_ as the product started to fall out of solution).

#### General procedures D: Demethylation with *p*-TsOH and LiCl

The respective methoxypyridine starting material (1.00 equiv.) was dissolved in *N*-methyl-2-pyrrolidone (NMP) (2 mL/100 mg) and transferred in a microwave vial. *p*-Toluenesulfonic acid (10 equiv.) and LiCl (10 equiv.) were added and the microwave tube was sealed. The reaction was stirred at 180°C until the reaction didn’t progress any further (reaction progress was monitored by LC-MS analysis).

#### General procedure E: *O*-Alkylation with methyl iodide

Sodium hydride (60% in mineral oil; 2.0 equiv.) was added to a suspension of the cyclohexanol starting material (1.0 equiv.) in dry CH_2_Cl_2_ (5 mL/100 mg). Iodomethane (2.0 equiv.) was added and the reaction mixture was stirred at room temperature. Reaction progression was monitored by LC-MS, in case of incomplete conversion another portion of sodium hydride (60% in mineral oil; 2.0 equiv.) and iodomethane (2.0 equiv.) was added and the reaction was stirred for another 24 h. When the reaction did not progress any further CH_2_Cl_2_ (150 mL) was added and the resulting organic layer was washed with water (2×100 mL), brine (100 mL) and then was dried with Na_2_SO_4_, filtered and the solvent removed under reduced pressure.

#### General procedure F: Oxidation Dess–Martin periodinane

The cyclohexanol starting material (1.0 equiv.) was suspended in CH_2_Cl_2_ (5 mL/100 mg) and Dess-Martin periodinane (2.0 equiv.) was added at 0°C. The reaction mixture was allowed to warm up to room temperature and stirred for 3 h. Reaction progression was monitored by LC-MS, in case of incomplete conversion another portion of Dess–Martin periodinane (2.0 equiv.) was added and the reaction was stirred for another 3 h. When complete reaction conversion was observed CH_2_Cl_2_ (150 mL) was added and the resulting organic layer was washed with 1 M NaOH (2×100 mL) and water (1 × 100 mL). The organic layer was dried with Na_2_SO_4_, filtered and concentrated under reduced pressure. 1-(2-Hydroxycyclohexyl)-4-(2-((4-(1-methyl-1*H*-pyrazol-4-yl)-benzyl) oxy)phenyl)pyridin-2(1*H*)-one (1). Synthesized as previously described in the literature.^[10a]^ 3-(2-Hydroxycyclohexyl)-6-(2-((4-(1-methyl-1*H*-pyrazol-4-yl)benzyl) oxy)phenyl)pyrimidin-4(3*H*)-one (2). Synthesized as previously described in the literature.^[10a]^

#### 2-((4-Bromopyridin-2-yl)oxy)cyclohexan-1-ol (4)

Synthesized as previously described in the literature.^[10a]^

#### 1-(2-Hydroxycyclohexyl)-4-(2-methoxy-5-methylphenyl)pyridin-2(1*H*)-one (5 a)

General procedure A. Purification by flash column chromatography (EtOAc 100%) yielded the titled product as a beige solid (978 mg, 85%). ^1^H NMR ([D_6_]DMSO): *δ*=7.65 (d, *J*=7.3 Hz, 1H), 7.22–7.18 (m, 1H), 7.17–7.14 (m, 1H), 7.02 (d, *J*=8.4 Hz, 1H), 6.44 (d, *J*=1.9 Hz, 1H), 6.36 (dd, *J*=7.2, 2.0 Hz, 1H), 4.75 (d, *J*=6.0 Hz, 1H), 4.63–4.45 (m, 1H), 3.86–3.74 (m, 4H), 2.28 (s, 3H), 2.05–1.97 (m, 1H), 1.78–1.67 (m, 3H), 1.63–1.48 (m, 1H), 1.42–1.28 (m, 3H); *m/z* MS (TOF ES^+^) 314.0 [*M*+H]^+^; LC-MS *t*_R_: 3.21.

#### 1-(2-Hydroxycyclohexyl)-4-(2-methoxy-4-methylphenyl)pyridin-2(1*H*)-one (5 b)

General procedure A. Purification by flash column chromatography (EtOAc 100%) yielded the titled product as a beige solid (230 mg, 67%). ^1^H NMR (CDCl_3_): *δ*=7.34 (d, *J*=7.3 Hz, 1H), 7.23 (d, *J*=7.7 Hz, 1H), 6.88–6.83 (m, 1H), 6.83–6.77 (m, 2H), 6.56 (dd, *J*=7.2, 1.9 Hz, 1H), 4.91–4.78 (m, 1H), 3.84 (s, 3H), 3.77–3.66 (m, 1H), 2.88 (br s, 1H), 2.41 (s, 3H), 2.30–2.20 (m, 1H), 2.05–1.96 (m, 1H), 1.92–1.80 (m, 2H), 1.74–1.35 (m, 4H); *m/z* MS (TOF ES^+^) 314.0 [*M*+H]^+^; LC-MS *t*_R_: 3.20.

#### 1-(2-Hydroxycyclohexyl)-4-(2-methoxy-4,5-dimethylphenyl)pyridin-2(1*H*)-one (5 c)

General procedure A. Purification by flash column chromatography (CH_2_Cl_2_ 100% CH_2_Cl_2_/MeOH 9:1), followed by a second flash column chromatography (EtOAc 100%) yielded the titled product as a white foam (201 mg, 56%). ^1^H NMR (CD_3_OD): *δ*=7.69–7.64 (m, 1H), 7.13 (s, 1H), 6.90 (s, 1H), 6.72–6.70 (m, 1H), 6.69–6.66 (m, 1H), 4.83–4.58 (m, 1H), 3.98–3.86 (m, 1H), 3.81 (s, 3H), 2.32 (s, 3H), 2.24 (s, 3H), 2.21–2.12 (m, 1H), 1.98–1.89 (m, 1H), 1.89–1.80 (m, 2H), 1.76–1.60 (m, 1H), 1.58–1.40 (m, 3H); *m/z* MS (TOF ES^+^) 328.0 [*M*+H]^+^; LC-MS *t*_R_: 3.38.

#### 1’-(2-Hydroxycyclohexyl)-4-methoxy-[3,4’-bipyridin]-2’(1’*H*)-one (5 d)

General procedure A. The desired product maintained in the water layer, therefore no work-up was performed. The reaction mixture was absorbed on silica and purified by flash column chromatography (CH_2_Cl_2_ 100% → CH_2_Cl_2_/MeOH_1_ 9:1) to afford the titled product as a yellow resin (497 mg, 90%). H NMR (CDCl_3_): *δ* = 8.41 (d, *J*=5.1 Hz, 1H), 8.28 (s, 1H), 7.34 (d, *J*=7.2 Hz, 1H), 6.81 (d, *J*=5.9 Hz, 1H), 6.62 (s, 1H), 6.37 (dd, *J*=7.2, 1.9 Hz, 1H), 4.72 (br t, *J*=9.4 Hz, 1H), 3.80 (s, 3H), 3.74–3.65 (m, 1H), 2.20–2.10 (m, 1H), 1.94–1.87 (m, 1H), 1.82–1.70 (m, 2H), 1.60–1.22 (m, 4H); *m/z* MS (TOF ES^+^) 301.0 [*M*+H]^+^; LC-MS *t*_R_: 2.85.

#### 1-(2-Hydroxycyclohexyl)-3’-methoxy-[4,4’-bipyridin]-2(1*H*)-one (5 e)

General procedure A. Purification by flash column chromatography (CH_2_Cl_2_ 100% →CH_2_Cl_2_/MeOH 9:1) yielded the titled product as a yellow resin (65 mg, 20%). Preparative HPLC (eluent 5–100%) of some impure fractions yielded another 25 mg (8%) of the titled product as a yellow resin. ^1^H NMR (CDCl_3_): *δ* = 8.38 (s, 1H), 8.32 (d, *J*=4.8 Hz, 1H), 7.40 (d, *J*=7.2 Hz, 1H), 7.22 (d, *J*=4.8 Hz, 1H), 6.77 (d, *J*=1.9 Hz, 1H), 6.48 (dd, *J*=7.2, 2.0 Hz, 1H), 4.86–4.77 (m, 1H), 3.94 (s, 3H), 3.78–3.69 (m, 1H), 2.27–2.18 (m, 1H), 2.03–1.95 (m, 1H), 1.90–1.80 (m, 2H), 1.69–1.32 (m, 4H) ; *m/z* MS (TOF ES^+^) 301.0 [*M*+H]^+^; LC-MS *t*_R_: 2.81.

#### 1’-(2-Hydroxycyclohexyl)-2-methoxy-[3,4’-bipyridin]-2’(1’H)-one (5 f)

General procedure A. Purification by flash column chromatography (CH_2_Cl_2_ 100% →CH_2_Cl_2_/MeOH 9:1) yielded the titled product as a light-brown foam (857 mg, 78%). ^1^H NMR (CDCl_3_): *δ* = 8.23 (dd, *J*=5.0, 1.9 Hz, 1H), 7.67 (dd, *J*=7.4, 1.9 Hz, 1H), 7.41 (d, *J*=7.2 Hz, 1H), 7.00 (dd, *J*=7.4, 5.0 Hz, 1H), 6.87 (d, *J*=1.8 Hz, 1H), 6.62 (dd, *J*=7.2, 2.0 Hz, 1H), 4.91–4.76 (m, 1H), 4.01 (s, 3H), 3.80–3.67 (m, 1H), 2.72 (br s, 1H), 2.32–2.18 (m, 1H), 2.10–1.98 (m, 1H), 1.93–1.82 (m, 2H), 1.75–1.31 (m, 4H); *m/z* MS (TOF ES^+^) 301.0 [*M*+H]^+^; LC-MS *t*_R_: 3.00.

#### 4-(2,6-Dimethoxyphenyl)-1-(2-hydroxycyclohexyl)pyridin-2(1*H*)-one (5 g)

General procedure A. Purification by flash column chromatography (CH_2_Cl_2_ 100% →CH_2_Cl_2_/MeOH 94:6), followed by recrystallization in EtOAc yielded the titled product as a white solid (446 mg, 37%) of. ^1^H NMR (CDCl_3_): *δ* = 7.37 (d, *J*=7.2 Hz, 1H), 7.32 (dd, *J*=8.4, 8.4 Hz, 1H), 6.77–6.72 (m, 1H), 6.64 (d, *J*=8.4 Hz, 2H), 6.37 (dd, *J*=7.1, 1.9 Hz, 1H), 4.90–4.80 (m, 1H), 3.78 (s, 6H), 3.76–3.69 (m, 1H), 3.16 (br s, 1H), 2.30–2.20 (m, 1H), 2.06–1.99 (m, 1H), 1.94–1.82 (m, 2H), 1.78–1.64 (m, 1H), 1.61–1.33 (m, 3H); *m/z* MS (TOF ES^+^) 330.0 [*M*+H]^+^; LC-MS *t*_R_: 3.14.

#### 1-(2-Hydroxycyclohexyl)-4-(3-methoxythiophen-2-yl)pyridin-2(1*H*)-one (5 h)

General procedure A. Purification by column chromatography (CH_2_Cl_2_ 100% → CH_2_Cl_2_/MeOH 9:1) yielded the titled product as a yellow oil (187 mg, 55%). ^1^H NMR (CDCl_3_): *δ* = 7.30 (d, *J*=7.3 Hz, 1H), 7.27 (d, *J*=5.6 Hz, 1H), 6.97 (d, *J*=2.0 Hz, 1H), 6.88 (d, *J*=5.6 Hz, 1H), 6.72 (dd, *J*=7.4, 2.1 Hz, 1H), 4.82–4.68 (m, 1H), 3.92 (s, 3H), 3.67 (td, *J*=10.5, 4.4 Hz, 1H), 2.26–2.15 (m, 1H), 1.98–1.87 (m, 1H), 1.86–1.75 (m, 2H), 1.66–1.29 (m, 4H); *m/z* MS (TOF ES^+^) 306.0 [*M*+H]^+^; LC-MS *t*_R_: 3.08.

### 4-(2-Hydroxy-5-methylphenyl)-1-(2-hydroxycyclohexyl)pyridin-2(1*H*)-one (6 a)

General procedure C. The desired compound was obtained as a yellow solid (550 mg, 59%). ^1^H NMR ([D_6_]DMSO): *δ* = 7.84 (d, *J*=7.2 Hz, 1H), 7.19–7.14 (m, 1H), 7.09–7.02 (m, 1H), 6.88 (d, *J*=8.2 Hz, 1H), 6.77–6.73 (m, 1H), 6.69 (dd, *J*=7.2, 2.0 Hz, 1H), 4.98 (s, 2H), 4.66–4.46 (m, 1H), 3.94–3.76 (m, 1H), 2.24 (s, 3H), 2.05–1.97 (m, 1H), 1.83–1.55 (m, 4H), 1.42–1.28 (m, 3H); *m/z* MS (TOF ES^+^) 300.0 [*M*+H]^+^; LC-MS *t*_R_: 3.07.

#### 1-(2-Hydroxycyclohexyl)-4-(2-methoxy-4-methylphenyl)pyridin-2(1*H*)-one (6 b)

General procedure C. The desired compound was obtained as a yellow-orange solid (220 mg, quantitative yield). ^1^H NMR ([D_6_]DMSO): *δ* = 9.75 (br s, 1H), 7.68 (d, *J*=7.3 Hz, 1H), 7.15 (d, *J*=7.8 Hz, 1H), 6.73–6.68 (m, 1H), 6.65–6.61 (m, 1H), 6.60 (d, *J*=1.9 Hz, 1H), 6.54 (dd, *J*=7.2, 2.0 Hz, 1H), 4.53–4.41 (m, 1H), 4.20 (br s, 1H), 3.80–3.66 (m, 1H), 2.17 (s, 3H), 1.98–1.88 (m, 1H), 1.71–1.45 (m, 4H), 1.33–1.18 (m, 3H); *m/z* MS (TOF ES^+^) 300.0 [*M*+H]^+^; LC-MS*t*_R_: 3.09.

#### 4-(2-Hydroxy-4,5-dimethylphenyl)-1-(2-hydroxycyclohexyl)pyridin-2(1*H*)-one (6 c)

General procedure C. The desired compound was obtained as a beige solid (192 mg, quantitative yield). ^1^H NMR ([D_6_]DMSO): *δ* = 9.42 (s, 1H), 7.64–7.58 (m, 1H), 7.13–7.04 (m, 1H), 6.79–6.70 (m, 1H), 6.57–6.52 (m, 1H), 6.51–6.46 (m, 1H), 4.66 (br s, 1H), 4.60–4.43 (m, 1H), 3.83–3.74 (m, 1H), 2.23–2.10 (m, 6H), 2.04–1.97 (m, 1H), 1.80–1.66 (m, 3H), 1.61–1.48 (m, 1H), 1.42–1.28 (m, 3H); *m/z* MS (TOF ES^+^) 314.0 [*M*+H]^+^; LC-MS *t*_R_: 3.17.

#### 4-Hydroxy-1’-(2-hydroxycyclohexyl)-[3,4’-bipyridin]-2’(1’*H*)-one (6 d)

General procedure D. Purification by flash column chromatography (CH_2_Cl_2_ 100% → CH_2_Cl_2_/MeOH 1:1) afforded the titled product as a brown oil (30 mg, 19%). ^1^H NMR (CD_3_OD): *δ* = 8.08 (d, *J*=1.6 Hz, 1H), 7.81 (dd, *J*=7.2, 1.6 Hz, 1H), 7.74 (d, *J*=7.2 Hz, 1H), 6.93 (d, *J*=1.9 Hz, 1H), 6.77 (dd, *J*=7.2, 2.0 Hz, 1H), 6.56 (d, *J*=7.2 Hz, 1H), 4.73 (br s, 1H), 3.94 (br s, 1H), 2.21–2.11 (m, 1H), 1.94–1.79 (m, 3H), 1.77–1.60 (m, 1H), 1.58–1.40 (m, 3H); *m/z* MS (TOF ES^+^) 286.9 [*M*+H]^+^; LC-MS *t*_R_: 1.71.

#### 3’-Hydroxy-1-(2-hydroxycyclohexyl)-[4,4’-bipyridin]-2(1*H*)-one (6 e)

General procedure D. Purification by preparative HPLC (eluent 5–100%) afforded the titled product as a light brown oil (22 mg, 11%). ^1^H NMR (CD_3_OD): *δ* = 8.11 (s, 1H), 8.01 (d, *J*=5.0 Hz, 1H), 7.68 (d, *J*=7.3 Hz, 1H), 7.30 (d, *J*=5.0 Hz, 1H), 6.79 (d, *J*=1.9 Hz, 1H), 6.65 (dd, *J*=7.2, 1.9 Hz, 1H), 4.66–4.57 (m, 1H), 3.84 (s, 1H), 2.08–2.02 (m, 1H), 1.85–1.68 (m, 3H), 1.67–1.56 (m, 1H), 1.46–1.32 (m, 3H); *m/z* MS (TOF ES^+^) 286.9 [*M*+H]^+^; LC-MS *t*_R_: 2.58.

#### Hydroxy-1’-(2-hydroxycyclohexyl)-[3,4’-bipyridin]-2’(1’*H*)-one

(6 d). General procedure D. Purification by flash column chromatography **(6 f)**. 1’-(2-Hydroxycyclohexyl)-2-methoxy-[3,4’-bipyridin]-2’(1’*H*)-one (445 mg, 148 mmol, 1.0 equiv.) was dissolved in ethanol (7 mL) and HBr (48% in water; 7 mL). The solution was stirred at 70°C for 3 h. The reaction mixture was cooled down to room temperature and saturated NaHCO_3_ was added until pH-9. The reaction mixture was evaporated to dryness and the residue was taken up in MeOH and adsorbed on silica gel. Purification by column chromatography (CH_2_Cl_2_ 100% CH_2_Cl_2_/MeOH 9:1) afforded the titled product as a beige foam (393 mg, 93%). ^1^H NMR ([D_6_]DMSO): *δ* = 11.93 (s, 1H), 7.80 (dd, *J*=7.0, 2.1 Hz, 1H), 7.63 (d, *J*=7.4 Hz, 1H), 7.48 (dd, *J*=6.4, 3Hz, 1H), 6.90 (d, *J*=2.0 Hz, 1H), 6.60 (dd, *J*=7.3, 2.1 Hz, 1H), 6.35–6.27 (m, 1H), 4.73 (d, *J*=6.0 Hz, 1H), 4.64–4.43 (m, 1H), 3.79 (br s, 1H), 2.07–1.94 (m, 1H), 1.82–1.64 (m, 3H), 1.63–1.45 (m, 1H), 1.41–1.24 (m, 3H); *m/z* MS (TOF ES^+^) 287.0 [*M*+H]^+^; LC-MS *t*_R_: 2.78.

#### 4-(2,6-Dihydroxyphenyl)-1-(2-hydroxycyclohexyl)pyridin-2(1*H*)-one (6 g)

General procedure C. The desired compound was obtained as a white solid (51 mg, 39%). ^1^H NMR ([D_6_]DMSO): *δ* = 9.38 (br s, 2H), 7.56 (d, *J*=7.2 Hz, 1H), 6.92 (t, *J*=8.1 Hz, 1H), 6.37 (d, *J*=8.1 Hz, 2H), 6.32 (d, *J*=1.8 Hz, 1H), 6.22 (dd, *J*=7.1, 1.9 Hz, 1H), 4.74 (br s, 1H), 4.63–4.42 (m, 1H), 3.89–3.73 (m, 1H), 2.07–1.94 (m, 1H), 1.81–1.63 (m, 3H), 1.62–1.45 (m, 1H), 1.43–1.21 (m, 3H); *m/z* MS (TOF ES^+^) 302.0 [*M*+H]^+^; LC-MS *t*_R_: 2.92.

#### 1-(2-Hydroxycyclohexyl)-4-(3-hydroxythiophen-2-yl)pyridin-2(1*H*)-one (6 h)

General procedure C. The desired compound was obtained as a yellow-orange resin (178 mg, quantitative yield). ^1^H NMR (CD_3_OD): *δ* = 8.08 (d, *J*=7.2 Hz, 1H), 7.50 (d, *J*=5.5 Hz, 1H), 7.43 (d, *J*=1.3 Hz, 1H), 7.34–7.30 (m, 1H), 6.74 (d, *J*=5.5 Hz, 1H), 4.66–4.54 (m, 1H), 3.96–3.88 (m, 1H), 2.15–2.03 (m, 1H), 1.97–1.87 (m, 1H), 1.82–1.62 (m, 3H), 1.47–1.31 (m, 3H); *m/z* MS (TOF ES^+^) 291.9 [*M*+H]^+^; LC-MS *t*_R_: 291.9.

#### 1-(2-Hydroxycyclohexyl)-4-(5-methyl-2-((4-(1-methyl-1*H*-pyrazol-4-yl)benzyl)oxy)phenyl)pyridin-2(1*H*)-one (7 a)

General procedure B. The residue was purified by flash column chromatography (PET/EtOAc 1:1 → EtOAc 100% → EtOAc/MeOH 9:1), followed by preparative HPLC (eluent 30–100%). The combined product fractions were taken up in CH_2_Cl_2_ and extracted with 1 M NaOH. The organic layer was dried with Na_2_SO_4_, filtered and the solvent was removed under reduced pressure. The titled product was obtained as a white resin (28 mg, 18%). ^1^H NMR (CDCl_3_): *δ* = 7.74 (d, *J*=0.6 Hz, 1H), 7.59 (s, 1H), 7.45–7.41 (m, 2H), 7.34–7.28 (m, 3H), 7.16–7.09 (m, 2H), 6.90 (d, *J*=8.3 Hz, 1H), 6.80–6.77 (m, 1H), 6.58 (dd, *J*=7.2, 2.0 Hz, 1H), 5.05 (s, 2H), 4.87–4.76 (m, 1H), 3.92 (s, 3H), 3.69 (td, *J*=10.5, 4.5 Hz, 1H), 2.67 (br s, 1H), 2.29 (s, 3H), 2.27–2.17 (m, 1H), 1.99–1.93 (m, 1H), 1.89–1.79 (m, 2H), 1.72–1.30 (m, 4H); *m/z* MS (TOF ES^+^) 469.9 [*M*+H]^+^; LC-MS *t*_R_: 3.38; HRMS: C_29_H_32_N_3_O_3_ [*M*+H]^+^ calcd 470.2444; found 470.2441.

### 1-(2-Hydroxycyclohexyl)-4-(4-methyl-2-((4-(1-methyl-1*H*-pyrazol-4-yl)benzyl)oxy)phenyl)pyridin-2(1*H*)-one (7 b)

General procedure B. The residue was purified by flash column chromatography (EtOAc 100% → EtOAc/MeOH 9:1) to afford the desired product as a white resin (45 mg, 29%). ^1^H NMR (CDCl_3_): *δ* = 7.74 (d, *J*=0.6 Hz, 1H), 7.58 (s, 1H), 7.46–7.41 (m, 2H), 7.35–7.31 (m, 2H), 7.28 (d, *J*=7.3 Hz, 1H), 7.25–7.20 (m, 1H), 6.86–6.81 (m, 2H), 6.80–6.77 (m, 1H), 6.58 (dd, *J*=7.2, 2.0 Hz, 1H), 5.06 (s, 2H), 4.86–4.75 (m, 1H), 3.91 (s, 3H), 3.68 (td, *J*=10.5, 4.5 Hz, 1H), 3.34–2.93 (br s, 1H), 2.35 (s, 3H), 2.24–2.15 (m, 1H), 2.00–1.90 (m, 1H), 1.87–1.76 (m, 2H), 1.66–1.31 (m, 4H); *m/z* MS (TOF ES^+^) 470.0 [*M*+H]^+^; LC-MS *t*_R_: 3.33; HRMS: C_29_H_32_N_3_O_3_ [*M*+H]^+^ calcd 470.2444; found 470.2464.

#### 4-(4,5-Dimethyl-2-((4-(1-methyl-1*H*-pyrazol-4-yl)benzyl)oxy) phenyl)-1-(2-hydroxycyclohexyl)pyridin-2(1*H*)-one (7 c)

General procedure A. The residue was purified by flash column chromatography (PET/EtOAc 1:1 → EtOAc 100% → EtOAc/MeOH 9:1), followed by preparative HPLC (eluent 30–100%). The combined product fractions were taken up in CH_2_Cl_2_ and washed with 1 M NaOH. The organic layer was dried with Na_2_SO_4_, filtered and the solvent was removed under reduced pressure. The titled product was obtained as a white resin (60 mg, 48%). ^1^H NMR (CDCl_3_): *δ* = 7.73 (d, *J*=0.6 Hz, 1H), 7.58 (s, 1H), 7.45–7.40 (m, 2H), 7.33–7.30 (m, 2H), 7.28 (d, *J*=7.3 Hz, 1H), 7.09 (s, 1H), 6.81 (s, 1H), 6.77 (d, *J*=1.8 Hz, 1H), 6.58 (dd, *J*=7.2, 2.0 Hz, 1H), 5.03 (s, 2H), 4.85–4.75 (m, 1H), 3.90 (s, 3H), 3.69 (td, *J*=10.7, 4.6 Hz, 1H), 3.15 (br s, 1H), 2.25 (s, 3H), 2.23–2.19 (m, 1H), 2.18 (s, 3H), 1.99–1.90 (m, 1H), 1.87–1.74 (m, 2H), 1.68–1.29 (m, 4H); *m/z* MS (TOF ES^+^) 484.0 [*M*+H]^+^; LC-MS *t*_R_: 3.38; HRMS: C_30_H_34_N_3_O_3_ [*M*+H]^+^ calcd 484.2600; found 484.2599.

#### 1’-(2-Hydroxycyclohexyl)-4-((4-(1-methyl-1*H*-pyrazol-4-yl)benzyl)oxy)-[3,4’-bipyridin]-2’(1’*H*)-one (7 d)

General procedure B. The desired product maintained in the water layer, therefore no work-up was performed. The residue was purified by flash column chromatography (CH_2_Cl_2_ 100% → CH_2_Cl_2_/MeOH 9:1), followed by preparative HPLC (eluent 5–100%) to give the desired product as a colourless resin (6 mg, 19%). ^1^H NMR (CD_3_OD): *δ* = 8.22 (d, *J*=2.3 Hz, 1H), 7.97 (s, 1H), 7.90 (dd, *J*=7.5, 2.3 Hz, 1H), 7.82 (d, *J*=0.6 Hz, 1H), 7.69 (d, *J*=7.2 Hz, 1H), 7.63–7.57 (m, 2H), 7.39–7.33 (m, 2H), 6.81 (d, *J*=1.9 Hz, 1H), 6.74 (dd, *J*=7.2, 1.9 Hz, 1H), 6.57 (d, *J*=7.4 Hz, 1H), 5.24 (s, 2H), 4.68 (br s, 1H), 3.91 (s, 3H), 3.91–3.83 (m, 1H), 2.18–2.09 (m, 1H), 1.91–1.77 (m, 3H), 1.74–1.59 (m, 1H), 1.54–1.39 (m, 3H); *m/z* MS (TOF ES^+^) 456.9 [*M*+H]^+^; LC-MS *t*_R_: 2.92; HRMS: C_27_H_29_N_4_O_3_ [*M*+H]^+^ calcd 457.2240; found 457.2244.

#### 1-(2-Hydroxycyclohexyl)-3’-((4-(1-methyl-1*H*-pyrazol-4-yl)benzyl) oxy)-[4,4’-bipyridin]-2(1*H*)-one (7 e)

General procedure B. The residue was purified by preparative HPLC (eluent 5–100%) to give the titled product as a colourless resin (4 mg, 11%). ^1^H NMR (CD_3_OD): *δ* = 8.51 (s, 1H), 8.29 (d, *J*=4.8 Hz, 1H), 7.97 (s, 1H), 7.82 (d, *J*=0.6 Hz, 1H), 7.77 (d, *J*=7.2 Hz, 1H), 7.58–7.53 (m, 2H), 7.46 (d, *J*=4.9 Hz, 1H), 7.43–7.38 (m, 2H), 6.82 (d, *J*=1.9 Hz, 1H), 6.70 (dd, *J*=7.2, 2.0 Hz, 1H), 5.29 (s, 2H), 4.75 (br s, 1H), 3.98–3.89 (m, 1H), 3.95 (s, 3H), 2.19–2.12 (m, 1H), 1.97–1.80 (m, 3H), 1.77–1.59 (m, 1H), 1.56–1.42 (m, 3H); *m/z* MS (TOF ES^+^) 456.9 [*M*+H]^+^; LC-MS *t*_R_: 2.47; HRMS: C_27_H_29_N_4_O_3_ [*M*+H]^+^ calcd 457.2240; found 457.2240.

#### 1’-(2-Hydroxycyclohexyl)-2-((4-(1-methyl-1*H*-pyrazol-4-yl)benzyl)oxy)-[3,4’-bipyridin]-2’(1’*H*)-one (7 f)

2-Hydroxy-1’-(2-hydroxycyclo-hexyl)-[3,4’-bipyridin]-2’(1’*H*)-one (60 mg, 210 μmol, 1.0 equiv.), Ag_2_CO_3_ (63.6 mg, 231 μmol, 1.1 equiv.), and 4-(4-(chloromethyl) phenyl)-1-methyl-1*H*-pyrazole (47.6 mg, 231 μmol, 1.1 equiv.) were stirred in DMF (4 mL) in a sealed microwave tube at 70°C (conventional heating) for 4 h, before EtOAc (150 mL) was added and the organic layer was washed with water (3 × 50 mL). The organic layer was dried with Na_2_SO_4_, filtered and the solvent was removed under reduced pressure. The residue was purified by flash column chromatography (CH_2_Cl_2_ 100% CH_2_Cl_2_/MeOH 92:8) to afford the titled product as a colourless oil (20 mg, 21%). ^1^H NMR (CDCl_3_): *δ* = 8.21 (dd, *J*=5.0, 1.9 Hz, 1H), 7.75 (s, 1H), 7.65 (dd, *J*=7.4, 1.9 Hz, 2H), 7.62 (s, 1H), 7.47–7.38 (m, 4H), 7.34 (d, *J*=7.3 Hz, 1H), 6.98 (dd, *J*=7.4, 5.0 Hz, 1H), 6.82 (d, *J*=1.8 Hz, 1H), 6.61 (dd, *J*=7.2, 1.9 Hz, 1H), 5.46 (s, 2H), 4.86–4.74 (m, 1H), 3.93 (s, 3H), 3.69 (td, *J*=10.5, 4.3 Hz, 1H), 2.27–2.15 (m, 1H), 2.01–1.91 (m, 1H), 1.89–1.80 (m, 2H), 1.72–1.30 (m, 4H); *m/z* MS (TOF ES^+^) 456.9 [*M*+H]^+^; LC-MS *t*_R_: 3.21; HRMS: C_27_H_29_N_4_O_3_ [*M*+H]^+^ calcd 457.2240; found 457.2240.

#### 4-(2-Hydroxy-6-((4-(1-methyl-1*H*-pyrazol-4-yl)benzyl)oxy)phenyl)-1-(2-hydroxycyclohexyl)pyridin-2(1*H*)-one (7 g)

General procedure B. The residue was purified by flash column chromatography (CH_2_Cl_2_ 100% → CH_2_Cl_2_/MeOH 9:1), followed by preparative HPLC (eluent 5–100%) to give the titled product as a white solid (3 mg, 2%). ^1^H NMR (CD_3_OD): *δ* = 7.94 (s, 1H), 7.80 (s, 1H), 7.68 (d, *J*=7.1 Hz, 1H), 7.53–7.48 (m, 2H), 7.34–7.28 (m, 2H), 7.16 (t, *J*=8.3 Hz, 1H), 6.69–6.63 (m, 2H), 6.57 (dd, *J*=8.2, 0.7 Hz, 1H), 6.54 (dd, *J*=7.1, 1.8 Hz, 1H), 5.06 (s, 2H), 4.81–4.66 (m, 1H), 3.05–3.90 (m, 1H), 3.93 (s, 3H), 2.21–2.14 (m, 1H), 2.00–1.93 (m, 1H), 1.88–1.81 (m, 2H), 1.79–1.61 (m, 1H), 1.56–1.42 (m, 3H); *m/z* MS (TOF ES^+^) 471.9 [*M*+H]^+^; LC-MS *t*_R_: 3.10; HRMS: C_28_H_30_N_3_O_4_ [*M*+H]^+^ calcd 472.2236; found 472.2229.

#### 1-(2-Hydroxycyclohexyl)-4-(3-((4-(1-methyl-1*H*-pyrazol-4-yl) benzyl)oxy)thiophen-2-yl)pyridin-2(1*H*)-one (7 h)

General procedure B. The residue was purified by flash column chromatography (CH_2_Cl_2_ 100% →CH_2_Cl_2_/MeOH 9:1), followed by preparative HPLC (eluent 30–100%) to give the titled product as a yellow oil (64 mg, 23%). ^1^H NMR (CDCl_3_): *δ* = 7.76 (d, *J*=0.7 Hz, 1H), 7.61 (s, 1H), 7.50–7.45 (m, 2H), 7.41–7.36 (m, 2H), 7.28 (d, *J*=7.4 Hz, 1H), 7.24 (d, *J*=5.6 Hz, 1H), 7.01 (d, *J*=2.0 Hz, 1H), 6.89 (d, *J*=5.6 Hz, 1H), 6.81 (dd, *J*=7.4, 2.1 Hz, 1H), 5.16 (s, 2H), 4.81–4.71 (m, 1H), 3.94 (s, 3H), 3.67 (td, *J*=10.5, 4.5 Hz, 1H), 3.05 (s, 1H), 2.25–2.16 (m, 1H), 1.97–1.90 (m, 1H), 1.86–1.77 (m, 2H), 1.65–1.25 (m, 4H); *m/z* MS (TOF ES^+^) 461.9 [*M*+H]^+^; LC-MS *t*_R_: 3.22; HRMS: C_26_H_28_N_3_O_3_S [*M*+H]^+^ calcd 462.1851; found 462.1865.

#### 4-(2-((4-Bromobenzyl)oxy)-4,5-dimethylphenyl)-1-(2-hydroxycy-clohexyl)pyridin-2(1*H*)-one (8 c)

General procedure B. The residue was purified by flash column chromatography (PET/EtOAc 1:1 EtOAc 100%) to afford the desired product as a white solid (140 mg, 51%). ^1^H NMR ([D_6_]DMSO): *δ* = 7.66–7.62 (m, 1H), 7.59–7.54 (m, 2H), 7.39–7.34 (m, 2H), 7.16 (s, 1H), 7.01 (s, 1H), 6.49–6.45 (m, 1H), 6.42 (dd, *J*=7.2, 2.0 Hz, 1H), 5.12 (s, 2H), 4.73 (d, *J*=6.0 Hz, 1H), 4.62–4.46 (m, 1H), 3.87–3.73 (m, 1H), 2.24 (s, 3H), 2.19 (s, 3H), 2.04–1.97 (m, 1H), 1.78–1.67 (m, 3H), 1.62–1.47 (m, 1H), 1.40–1.24 (m, 4H); *m/z* MS (TOF ES^+^) 481.8 [*M*+H]^+^; LC-MS *t*_R_: 3.60.

#### 1’-(2-Hydroxycyclohexyl)-1-(4-(1-methyl-1*H*-pyrazol-4-yl)benzyl)-[3,4’-bipyridine]-2,2’(1*H*,1’*H*)-dione (9)

General procedure B. The residue was purified by flash column chromatography (CH_2_Cl_2_ 100% → CH_2_Cl_2_/MeOH 92:8) to afford the title compound as a white foam (18 mg, 23%). Remark: The O-alkylated isomer **7f** was formed as the minor product and was isolated as a colourless oil (1 mg, 1%). ^1^H NMR (CDCl_3_): *δ* = 7.74 (d, *J*=0.6 Hz, 1H), 7.61 (s, 1H), 7.56 (dd, *J*=7.0, 2.0 Hz, 1H), 7.48–7.41 (m, 3H), 7.40–7.33 (m, 3H), 6.90 (dd, *J*=7.2, 2.0 Hz, 1H), 6.85 (d, *J*=1.9 Hz, 1H), 6.28 (t, *J*=6.9 Hz, 1H), 5.21 (d, *J*=14.3 Hz, 1H), 5.15 (d, *J*=14.4 Hz, 1H), 4.88–4.75 (m, 1H), 3.95 (s, 3H), 3.77–3.69 (m, 1H), 2.79 (br s, 1H), 2.27–2.18 (m, 1H), 2.00–1.92 (m, 1H), 1.91–1.80 (m, 2H), 1.72–1.60 (m, 1H), 1.59–1.33 (m, 3H); *m/z* MS (TOF ES^+^) 456.9 [*M*+H]^+^; LC-MS *t*_R_: 3.02; HRMS: C_27_H_29_N_4_O_3_ [*M*+H]^+^ calcd 457.2240; found 457.2238.

#### 4-Bromo-1-cyclohexylpyridin-2(1*H*)-one (11 b)

A mixture of 4-bromo-2-hydroxypyridine (500 mg, 2.87 mmol, 1.00 equiv.), bromo-cyclohexane (1.42 mL, 11.5 mmol, 4.0 equiv.), K_2_CO_3_ (874 mg, 6.32 mmol, 2.2 equiv.) was stirred at 120°C for 5 days. The reaction mixture was cooled to room temperature and volatile compounds were removed under reduced pressure. The residue was taken up in EtOAc and extracted with water (2 × 75 mL) and brine (75 mL). The organic layer was dried with Na_2_SO_4_ and filtered, before concentration under reduced pressure. Purification by flash column chromatography (CH_2_Cl_2_ 100% → CH_2_Cl_2_/MeOH 9:1) yielded the titled product as a yellow oil (118 mg, 16%). ^1^H NMR (CDCl_3_): *δ* = 7.95 (d, *J*=5.5 Hz, 1H), 6.96 (dd, *J*=5.5, 1.7 Hz, 1H), 6.92–6.87 (m, 1H), 5.05–4.97 (m, 1H), 2.03–1.92 (m, 2H), 1.82–1.73 (m, 2H), 1.61–1.24 (m, 6H); *m/z* MS (TOF ES^+^) 255.8 [*M*+H]^+^; LC-MS *t*_R_: 3.90.

#### 2-(4-Bromo-2-oxopyridin-1(2*H*)-yl)-*N,N*-dimethylacetamide (11 c)

A mixture of 4-bromo-2-hydroxypyridine (300 mg, 1.72 mmol, 1.00 equiv.), 2-bromo-*N,N*-dimethylacetamide (572 mg, 3.45 mmol, 2.0 equiv.), K_2_CO_3_ (477 mg, 3.45 mmol, 2.0 equiv.) and KI (28.6 mg, 172 μmol, 0.1 equiv.) was stirred at room temperature for 24 h before EtOAc was added. The organic layer was washed with water (2×75 mL) and brine (75 mL) and then dried with Na_2_SO_4_ and filtered, before it was concentrated under reduced pressure. Purification by flash column chromatography (CH_2_Cl_2_ 100% CH_2_Cl_2_/MeOH 9:1) yielded the desired product as a white solid (53 mg, 12%). The water layer was reduced and the residue was purified by flash column chromatography (CH_2_Cl_2_ 100% CH_2_Cl_2_/MeOH 9:1) to give an additional 380 mg (85%) of the titled product. ^1^H NMR (CDCl_3_): *δ* = 6.99 (d, *J*=7.3 Hz, 1H), 6.64 (d, *J*=2.1 Hz, 1H), 6.19 (dd, *J*=7.3, 2.2 Hz, 1H), 4.53 (s, 2H), 2.96 (s, 3H), 2.81 (s, 3H); *m/z* MS (TOF ES^+^) 215.8 [*M*-N(CH_3_)_2_ +H]^+^; LC-MS *t*_R_: 1.50.

#### 4-Bromo-1-(2-oxocyclohexyl)pyridin-2(1*H*)-one (11 d)

General procedure F. The titled product was obtained as a white solid (474 mg, 96%). The crude material was used in the next step without further purification. ^1^H NMR (CDCl_3_): *δ* = 7.00 (d, *J*=7.4 Hz, 1H), 6.83 (d, *J*=2.1 Hz, 1H), 6.37 (dd, *J*=7.4, 2.2 Hz, 1H), 5.78–5.66 (m, 1H), 2.65–2.48 (m, 2H), 2.35–2.28 (m, 1H), 2.24–2.15 (m, 1H), 2.13–2.04 (m, 1H), 2.01–1.83 (m, 2H), 1.81–1.69 (m, 1H); *m/z* MS (TOF ES^+^) 269.8 [*M*+H]^+^; LC-MS *t*_R_: 3.01.

1-(2-Hydroxycyclohexyl)-4-(2-hydroxyphenyl)pyridin-2(1*H*)-one (12a). Synthesized as previously described in the literature.^[10a,14]^

#### 1-Cyclohexyl-4-(2-hydroxyphenyl)pyridin-2(1*H*)-one (12 b)

General procedure A. Purification by flash column chromatography (PET/EtOAc 8:2 → EtOAc 100%) yielded the desired product as a beige solid (82 mg, 66%). ^1^H NMR (CDCl_3_): *δ* = 8.22–8.17 (m, 1H), 7.31–7.25 (m, 2H), 7.06–6.96 (m, 3H), 6.91–6.87 (m, 1H), 6.17 (br s, 1H), 5.09–4.99 (m, 1H), 2.06–2.00 (m, 2H), 1.84–1.74 (m, 2H), 1.63–1.29 (m, 6H); *m/z* MS (TOF ES^+^) 270.0 [*M*+H]^+^; LC-MS *t*_R_: 3.51.

#### 2-(4-(2-Hydroxyphenyl)-2-oxopyridin-1(2*H*)-yl)-*N,N*-dimethylacetamide (12 c)

General procedure A. Purification by flash column chromatography (CH_2_Cl_2_ 100% → CH_2_Cl_2_/MeOH 9:1) yielded the desired product as a beige solid (70 mg, 29%). ^1^H NMR (CD_3_OD): *δ* = 7.37 (d, *J*=7.1 Hz, 1H), 7.25–7.18 (m, 1H), 7.16–7.08 (m, 1H), 6.83–6.77 (m, 2H), 6.74–6.70 (m, 1H), 6.65–6.58 (m, 1H), 4.74 (s, 2H), 3.02 (s, 3H), 2.85 (s, 3H); *m/z* MS (TOF ES^+^) 272.9 [*M*+H]^+^; LC-MS *t*_R_: 2.84.

#### 4-(2-Hydroxyphenyl)-1-(2-oxocyclohexyl)pyridin-2(1*H*)-one (12 d)

General procedure A. Purification by flash column chromatography (EtOAc 100%) yielded the titled product as a beige solid (82 mg, 14%). ^1^H NMR ([D_6_]DMSO): *δ* = 9.90 (s, 1H), 7.52 (d, *J*=7.2 Hz, 1H), 7.34 (dd, *J*=7.7, 1.6 Hz, 1H), 7.27–7.20 (m, 1H), 6.97 (dd, *J*=8.2, 0.9 Hz, 1H), 6.89 (td, *J*=7.5, 1.1 Hz, 1H), 6.58 (d, *J*=1.8 Hz, 1H), 6.49 (dd, *J*=7.2, 2.0 Hz, 1H), 5.54 (dd, *J*=12.6, 6.4 Hz, 1H), 2.64 (td, *J*=13.9, 6.3 Hz, 1H), 2.47–2.37 (m, 1H), 2.24–1.68 (m, 6H); *m/z* MS (TOF ES^+^) 283.9 [*M*+H]^+^; LC-MS *t*_R_: 3.05.

#### 1-Cyclohexyl-4-(2-((4-(1-methyl-1*H*-pyrazol-4-yl)benzyl)oxy) phenyl)pyridin-2(1*H*)-one (13 b)

General procedure B. Purification by flash column chromatography (PET/EtOAc 1:1 → EtOAc 100%), followed by preparative HPLC (eluent 30–100%). The combined product fractions were taken up in CH_2_Cl_2_ and extracted with 1 M NaOH. The organic layer was dried with Na_2_SO_4_, filtered and the solvent was removed under reduced pressure. The residue was further purified by flash column chromatography (CH_2_Cl_2_ 100% CH_2_Cl_2_/MeOH 9:1) to afford the titled product as a colourless oil (20 mg, 15%). Remark: Product was only 90% pure according to ^1^H NMR. ^1^H NMR (CDCl_3_): *δ* = 8.14 (d, *J*=5.3 Hz, 1H), 7.74 (s, 1H), 7.57 (s, 1H), 7.45–7.41 (m, 2H), 7.38–7.30 (m, 4H), 7.10–7.01 (m, 3H), 6.94–6.91 (m, 1H), 5.12–5.01 (m, 3H), 3.92 (s, 3H), 2.09–1.97 (m, 2H), 1.85–1.75 (m, 2H), 1.63–1.25 (m, 6H); *m/z* MS (TOF ES^+^) 440.0 [*M*+H]^+^; LC-MS *t*_R_: 3.84; HRMS: C_28_H_30_N_3_O_2_ [*M*+H]^+^ calcd 440.2338; found 440.2344.

#### *N,N*-Dimethyl-2-(4-(2-((4-(1-methyl-1*H*-pyrazol-4-yl)benzyl)oxy) phenyl)-2-oxopyridin-1(2*H*)-yl)acetamide (13 c)

General procedure B. The residue was purified by flash column chromatography (CH_2_Cl_2_ 100% → CH_2_Cl_2_/MeOH 9:1) to afford the titled product as a colourless oil (83 mg, 72%). ^1^H NMR (CDCl_3_): *δ* = 7.75 (d, *J*=0.7 Hz, 1H), 7.60 (s, 1H), 7.48–7.43 (m, 2H), 7.37–7.31 (m, 4H), 7.28–7.24 (m, 1H), 7.05–7.00 (m, 2H), 6.78–6.75 (m, 1H), 6.53 (dd, *J*=7.1, 1.9 Hz, 1H), 5.10 (s, 2H), 4.76 (s, 2H), 3.92 (s, 3H), 3.15 (s, 3H), 2.99 (s, 3H); *m/z* MS (TOF ES^+^) 442.9 [*M*+H]^+^; LC-MS *t*_R_: 3.16; HRMS: C_26_H_27_N_4_O_3_ [*M*+H]^+^ calcd 443.2083; found 433.2083.

#### 4-(2-((4-(1-Methyl-1*H*-pyrazol-4-yl)benzyl)oxy)phenyl)-1-(2-oxocy-clohexyl)pyridin-2(1*H*)-one (13 d)

General procedure B. The residue was purified by flash column chromatography (EtOAc 100% → EtOAc/MeOH 9:1), followed by preparative HPLC (eluent 30–100%). The combined product fractions were taken up in CH_2_Cl_2_ and extracted with 1 M NaOH. The organic layer was dried with Na_2_SO_4_, filtered and the solvent was removed under reduced pressure. The titled product was obtained as a colourless oil (80 mg, 71%). ^1^H NMR (CDCl_3_): *δ* = 7.73 (d, *J*=0.6 Hz, 1H), 7.58 (s, 1H), 7.46–7.41 (m, 2H), 7.36–7.28 (m, 4H), 7.10 (d, *J*=7.2 Hz, 1H), 7.03–6.98 (m, 2H), 6.77 (d, *J*=1.7 Hz, 1H), 6.51 (dd, *J*=7.2, 2.0 Hz, 1H), 5.81 (dd, *J*=12.1, 6.0 Hz, 1H), 5.09 (s, 2H), 3.90 (s, 3H), 2.67–2.48 (m, 2H), 2.38–2.30 (m, 1H), 2.25–2.12 (m, 1H), 2.12–1.88 (m, 3H), 1.81–1.67 (m, 1H); *m/z* MS (TOF ES^+^) 454.0 [*M*+H]^+^; LC-MS *t*_R_: 3.32; HRMS: C_28_H_28_N_3_O_3_ [*M*+H]^+^ calcd 454.2131; found 454.2152.

#### 1-(2-Methoxycyclohexyl)-4-(2-((4-(1-methyl-1*H*-pyrazol-4-yl) benzyl)oxy)phenyl)pyridin-2(1*H*)-one (14)

General procedure E. The residue was adsorbed on silica gel and purified by flash column chromatography (CH_2_Cl_2_ 100% → CH_2_Cl_2_/MeOH 9:1) to give the titled product as a colourless oil (13 mg, 25%). ^1^H NMR (CDCl_3_): *δ* = 7.74 (d, *J*=0.6 Hz, 1H), 7.60 (s, 1H), 7.46–7.41 (m, 2H), 7.39–7.30 (m, 4H), 7.22 (d, *J*=7.2 Hz, 1H), 7.07–7.00 (m, 2H), 6.77 (d, *J*=1.8 Hz, 1H), 6.51 (dd, *J*=7.2, 2.0 Hz, 1H), 5.10 (s, 2H), 4.90–6.60 (m, 1H), 3.94 (s, 3H), 3.65–3.45 (m, 1H), 3.22 (s, 3H), 2.34–2.23 (m, 1H), 2.05–1.98 (m, 1H), 1.96–1.72 (m, 3H), 1.52–1.28 (m, 3H); *m/z* MS (TOF ES^+^) 469.9 [*M*+H]^+^; LC-MS *t*_R_: 3.42; HRMS: C_29_H_32_N_3_O_3_ [*M*+H]^+^ calcd 470.2444; found 470.2455.

#### 2,2“-Dimethoxy-1,1’:4’,1“-terphenyl (16)

PdCl_2_(PPh_3_)_2_ (298 mg, 424 μmol, 0.2 equiv.) was added to a mixture of 1,4-dibromobenzene (500 mg, 2.12 mmol, 1.0 equiv.) and (2-methoxyphenyl)boronic acid (805 mg, 5.30 mmol, 2.5 equiv.) in degassed (by sonication followed by a stream of nitrogen) THF/1 M Na_2_CO_3(aq)_ (3:1, 16 mL) flushed with nitrogen. The reaction mixture was stirred at reflux for 4 h before the THF was evaporated under reduced pressure. The mixture was diluted with water (50 mL) and extracted with EtOAc (3×50 mL). The combined organic layers were washed with brine (50 mL), dried over Na_2_SO_4_ and filtered, before concentration under reduced pressure. Purification by flash column chromatography (PET 100% PET/EtOAc 8:2) yielded the desired product as a yellow solid (420 mg, 68%). ^1^H NMR (CDCl_3_): *δ* = 7.59 (s, 4H), 7.39 (dd, *J*=7.5, 1.7 Hz, 2H), 7.33 (ddd, *J*=8.2, 7.5, 1.8 Hz, 2H), 7.05 (td, *J*=7.5, 1.1 Hz, 2H), 7.02–6.99 (m, 2H), 3.85 (s, 6H); LC-MS *t*_R_: 3.81, no ionization was observed.

#### [1,1’:4’,1“-Terphenyl]-2,2“-diol (17)

General procedure C. The desired compound was obtained as a beige solid (192 mg, quantitative yield). ^1^H NMR (CDCl_3_): *δ* = 7.51 (s, 4H), 7.22–7.15 (m, 4H), 6.97–6.87 (m, 4H); *m/z* MS (TOF ES^+^) 261.1 [*M* H]^-^; LC-MS *t*_R_: 3.48.

#### 2“-((4-Bromobenzyl)oxy)-[1,1’:4’,1“-terphenyl]-2-ol (18)

General procedure B. The residue was purified by flash column chromatography (PET 100% PET/EtOAc 8:2) to afford the desired product as a white resin (103 mg, 36%). ^1^H NMR (CDCl_3_): *δ* = 7.74–7.69 (m, 2H), 7.57–7.53 (m, 2H), 7.51–7.42 (m, 4H), 7.36–7.32 (m, 2H), 7.27–7.21 (m, 2H), 7.16–7.09 (m, 2H), 7.08–7.02 (m, 2H), 5.09 (s, 2H); LC-MS *t*_R_: 3.83, no ionization was observed.

#### 2“-((4-(1-Methyl-1*H*-pyrazol-4-yl)benzyl)oxy)-[1,1’:4’,1“-terphenyl]-2-ol (19)

General procedure A. The residue was purified by flash column chromatography (CH_2_Cl_2_ 100% → CH_2_Cl_2_/MeOH 8:2) and recrystallization in DMF yielded the desired product as a white solid (26 mg, 25%). ^1^H NMR ([D_6_]DMSO): *δ* = 9.62 (s, 1H), 8.17 (s, 1H), 7.90 (s, 1H), 7.65 (m, 4H), 7.63–7.59 (m, 2H), 7.47–7.41 (m, 3H), 7.40–7.34 (m, 2H), 7.28–7.25 (m, 1H), 7.25–7.20 (m, 1H), 7.15–7.09 (m, 1H), 7.05–7.00 (m, 1H), 6.98–6.92 (m, 1H), 5.21 (s, 2H), 3.90 (s, 3H); *m/z* MS (TOF ES^+^) 432.9 [*M* H]^+^; LC-MS *t*_R_: 3.64; HRMS: C_29_H_25_N_2_O_2_ [*M*+H]^+^ calcd 433.1916; found 433.1918.

#### 6-(2,6-Dimethoxyphenyl)pyrimidin-4(3*H*)-one (21)

General procedure A. No work-up, the reaction mixture was absorbed on silica gel and purified by flash column chromatography (CH_2_Cl_2_ 100% → CH_2_Cl_2_/MeOH 9:1), followed by a second flash column chromatography (PET/EtOAc 1:1 EtOAc 100%) to afford the desired product as a white foam (631 mg, 18%). ^1^H NMR (CDCl_3_): *δ* = 13.19 (br s, 1H), 8.24 (d, *J*=1.0 Hz, 1H), 7.34 (t, *J*=8.4 Hz, 1H), 6.64 (d, *J*=8.4 Hz, 2H), 6.55 (d, *J*=1.0 Hz, 1H), 3.79 (s, 6H); *m/z* MS (TOF ES^+^) 233.0 [*M*+H]^+^; LC-MS *t*_R_: 2.74.

6-Bromo-3-((1*S*,2*S*)-2-hydroxycyclohexyl)pyrimidin-4(3*H*)-one (23). Synthesized as previously described in the literature.^[14]^

#### 3-(2-Hydroxycyclohexyl)-6-(2-methoxypyridin-3-yl)pyrimidin-4(3*H*)-one (24 a)

General procedure A. Purification by flash column chromatography (CH_2_Cl_2_ 100%!CH_2_Cl_2_/MeOH 9:1), followed by flash column chromatography (PET/EtOAc 1:1→ EtOAc _1_ 100%) yielded the titled product as a grey solid (250 mg, 40%). H NMR ([D_6_]DMSO): *δ* = 8.57 (s, 1H), 8.42 (dd, *J*=7.6, 2.0 Hz, 1H), 8.29 (dd, *J*=4.9, 2.0 Hz, 1H), 7.17 (dd, *J*=7.6, 4.9 Hz, 1H), 7.09 (s, 1H), 4.97 (d, *J*=5.7 Hz, 1H), 4.32 (br s, 1H), 4.00 (s, 3H), 4.00–3.90 (m, 1H), 2.06–1.97 (m, 1H), 1.86–1.66 (m, 4H), 1.39–1.25 (m, 3H); *m/z* MS (TOF ES^+^) 301.9 [*M*+H]^+^; LC-MS *t*_R_: 3.02.

#### 3-(2-Hydroxycyclohexyl)-6-(3-methoxythiophen-2-yl)pyrimidin-4(3*H*)-one (24 b)

General procedure A. Purification by flash column chromatography (PET/EtOAc 1:1 → EtOAc 100%) yielded the desired compound as a light yellow oil/foam (182 mg, 46%). ^1^H NMR (CDCl_3_): *δ* = 8.07 (s, 1H), 7.35 (d, *J*=5.5 Hz, 1H), 6.97 (s, 1H), 6.83 (d, *J*=5.6 Hz, 1H), 4.57 (br s, 1H), 4.52–4.37 (m, 1H), 3.95 (br s, 1H), 3.90 (s, 3H), 2.26–2.17 (m, 1H), 2.00–1.91 (m, 1H), 1.86–1.62 (m, 3H), 1.57–1.33 (m, 3H); *m/z* MS (TOF ES^+^) 306.9 [*M*+H]^+^; LC-MS *t*_R_: 3.07.

#### 6-(2,6-Dimethoxyphenyl)-3-(2-hydroxycyclohexyl)pyrimidin-4(3*H*)-one (24 c)

A mixture of 6-(2,6-dimethoxyphenyl)pyrimidin-4(3*H*)-one (**21**) (631 mg, 2.72 mmol, 1.0 equiv.), 1,2-cyclohexene oxide (2.75 mL, 27.2 mmol, 10.0 equiv.), and K_2_CO_3_ (939 mg, 6.79 mmol, 2.5 equiv.) was stirred at 120°C for 22 h. The reaction mixture was cooled to room temperature and concentrated to dryness under reduced pressure. Residue was adsorbed on silica gel and purified by flash column chromatography (CH_2_Cl_2_ 100% CH_2_Cl_2_/MeOH 9:1) to afford the titled product as a white solid (460 mg, 51%). ^1^H NMR ([D_6_]DMSO): *δ* = 8.46 (s, 1H), 7.35 (t, *J*=8.4 Hz, 1H), 6.74 (d, *J*=8.5 Hz, 2H), 6.21 (d, *J*=0.5 Hz, 1H), 4.97 (d, *J*=6.0 Hz, 1H), 4.54–4.20 (m, 1H), 3.98 (br s, 1H), 3.71 (s, 6H), 2.08–1.98 (m, 1H), 1.93–1.59 (m, 4H), 1.43–1.24 (m, 3H); *m/z* MS (TOF ES^+^) 330.9 [*M*+H]^+^; LC-MS *t*_R_: 3.20.

#### 3-(2-Hydroxycyclohexyl)-6-(2-hydroxypyridin-3-yl)pyrimidin-4(3*H*)-one (25 a)

3-(2-Hydroxycyclohexyl)-6-(2-methoxypyridin-3-yl) pyrimidin-4(3*H*)-one (250 mg, 830 mmol, 1.0 equiv.) was dissolved in ethanol (7 mL) and HBr (48% in water; 7 mL). The solution was stirred at 70°C for 1 h. The reaction mixture was cooled down to room temperature and the ethanol was removed under reduced pressure. CH_2_Cl_2_ and saturated NaHCO_3_ were added until pH 9. The product that went into solution was transferred to a separation funnel. The layers were separated and the organic layer was combined with the insoluble solid after the work up. The CH_2_Cl_2_ was removed under reduced pressure and the obtained residue was dried under high vacuum. The title compound was afforded as a light-yellow solid (238 mg, quantitative yield). ^1^H NMR (CD_3_OD): *δ* = 8.95 (s, 1H), 8.60 (dd, *J*=7.4, 2.1 Hz, 1H), 7.76 (dd, *J*=6.3, 2.0 Hz, 1H), 7.53 (s, 1H), 6.67 (dd, *J*=7.4, 6.4 Hz, 1H), 4.46 (br s, 1H), 4.06 (br s, 1H), 2.22–2.16 (m, 1H), 2.08–2.02 (m, 1H), 1.97–1.82 (m, 3H), 1.54–1.42 (m, 3H); *m/z* MS (TOF ES^+^) 287.9 [*M*+H]^+^; LC-MS *t*_R_: 2.81.

#### 3-(2-Hydroxycyclohexyl)-6-(3-hydroxythiophen-2-yl)pyrimidin-4(3*H*)-one (25 b)

General procedure C. An additional 2.0 equiv. of BBr_3_ were added. Isolated was an orange oil (127 mg, 73%) containing 80% of the desired product and 20% of starting material. The isolated mixture was used in the next step without further purification. ^1^H NMR (CDCl_3_): *δ* = 8.16 (s, 1H), 7.19 (d, *J*=5.4 Hz, 1H), 6.63 (d, *J*=5.4 Hz, 1H), 6.10 (s, 1H), 4.55–4.35 (m, 2H), 3.89 (br s, 1H), 2.25–2.17 (m, 1H), 2.03–1.92 (m, 1H), 1.86–1.62 (m, 3H), 1.54–1.33 (m, 3H), *m/z* MS (TOF ES^-^) 291.0 [*M*+H]^+^; LC-MS *t*_R_: 3.07.

#### 6-(2-Hydroxy-6-methoxyphenyl)-3-(2-hydroxycyclohexyl)pyrimidin-4(3*H*)-one (25 c)

General procedure C. Purification by flash column chromatography (PET/EtOAC 2:8→EtOAc 100%) _1_ yielded the titled product as a light yellow oil (40 mg, 87%). H NMR (CDCl_3_): *δ* = 13.28 (br s, 1H), 8.18 (s, 1H), 7.34 (d, *J*=0.6 Hz, 1H), 7.14 (t, *J*=8.3 Hz, 1H), 6.49 (dd, *J*=8.3, 1.1 Hz, 1H), 6.34 (dd, *J*=8.3, 0.9 Hz, 1H), 4.50–4.36 (m, 1H), 3.96–3.85 (m, 1H), 3.78 (s, 3H), 3.73 (s, 1H), 2.24–2.17 (m, 1H), 2.02–1.96 (m, 1H), 1.87–1.79 (m, 2H), 1.76–1.64 (m, 1H), 1.51–1.36 (m, 3H); *m/z* MS (TOF ES^+^) 316.9 [*M*+H]^+^; LC-MS *t*_R_: 3.25.

#### 6-(2,6-Dihydroxyphenyl)-3-(2-hydroxycyclohexyl)pyrimidin-4(3*H*)-one (25 d)

General procedure D. Purification by flash column chromatography (CH_2_Cl_2_ 100% → CH_2_Cl_2_/MeOH 9:1) to afford the titled product as a beige solid (30 mg, 34%). ^1^H NMR ([D_6_]DMSO): *δ* = 11.74 (s, 2H), 8.70 (s, 1H), 7.31 (s, 1H), 7.08 (t, *J*=8.2 Hz, 1H), 6.39 (d, *J*=8.2 Hz, 2H), 5.03 (d, *J*=5.6 Hz, 1H), 4.48–4.22 (m, 1H), 4.01–3.86 (m, 1H), 2.06–1.97 (m, 1H), 1.90–1.63 (m, 4H), 1.40–1.27 (m, 3H); *m/z* MS (TOF ES^+^) 302.9 [*M*+H]^+^; LC-MS *t*_R_: 2.96.

#### 3-(2-Hydroxycyclohexyl)-6-(2-((4-(1-methyl-1*H*-pyrazol-4-yl) benzyl)oxy)pyridin-3-yl)pyrimidin-4(3*H*)-one (26 a)

3-(2-Hydroxy-cyclohexyl)-6-(2-hydroxypyridin-3-yl)pyrimidin-4(3*H*)-one (**25a**) (60 mg, 209 μmol, 1.0 equiv.), Ag_2_CO_3_ (63.3 mg, 230 μmol, 1.1 equiv.), and 4-(4-(chloromethyl)phenyl)-1-methyl-1*H*-pyrazole (47.5 mg, 230 μmol, 1.1 equiv.) were stirred in DMF (3 mL) in a sealed microwave tube at 70°C (conventional heating) for 4 h, before EtOAc (150 mL) was added and the organic layer was washed with water (3×50 mL). The organic layer was dried with Na_2_SO_4_, filtered and the solvent was removed under reduced pressure. The residue was purified by flash column chromatography (CH_2_Cl_2_ 100% → CH_2_Cl_2_/MeOH 9:1), followed by preparative HPLC (eluent 5–100%) to afford the titled product as a colourless oil (7 mg, 7%). ^1^H NMR (CDCl_3_): *δ* = 8.45 (dd, *J*=7.6, 2.0 Hz, 1H), 8.24 (dd, *J*=4.9, 2.0 Hz, 1H), 8.23 (s, 1H), 7.76 (d, *J*=0.6 Hz, 1H), 7.61 (s, 1H), 7.50–7.45 (m, 4H), 7.42 (s, 1H), 7.04 (dd, *J*=7.6, 4.9 Hz, 1H), 5.57 (s, 2H), 4.56–4.46 (m, 1H), 3.95 (s, 3H), 3.95–3.86 (m, 1H), 2.27–2.21 (m, 1H), 2.14 (br s, 1H), 2.05–1.99 (m, 1H), 1.94–1.78 (m, 3H), 1.56–1.36 (m, 3H); *m/z* MS (TOF ES^+^) 457.9 [*M*+H]^+^; LC-MS *t*_R_: 3.32; HRMS: C_26_H_28_N_5_O_3_ [*M*+H]^+^ calcd 458.2192; found 458.2188.

#### 3-(2-Hydroxycyclohexyl)-6-(3-((4-(1-methyl-1*H*-pyrazol-4-yl) benzyl)oxy)thiophen-2-yl)pyrimidin-4(3*H*)-one (26 b)

General procedure B. Purification by flash column chromatography (CH_2_Cl_2_ 100% → CH_2_Cl_2_/MeOH 9:1), followed by preparative HPLC (eluent 5–100%) yielded the titled product as a white resin (74 mg, 47%). ^1^H NMR (CDCl_3_): *δ* = 8.04 (s, 1H), 7.71 (d, *J*=0.5 Hz, 1H), 7.55 (s, 1H), 7.45–7.39 (m, 2H), 7.36–7.31 (m, 2H), 7.28 (d, *J*=5.6 Hz, 1H), 7.12 (s, 1H), 6.81 (d, *J*=5.6 Hz, 1H), 5.14 (s, 2H), 4.47–4.34 (m, 1H), 3.89 (s, 3H), 3.88–3.78 (m, 1H), 3.33 (br s, 1H), 2.22–2.11 (m, 1H), 1.94–1.88 (m, 1H), 1.84–1.64 (m, 3H), 1.51–1.28 (m, 3H); *m/z* MS (TOF ES^+^) 462.8 [*M*+H]^+^; LC-MS *t*_R_: 3.25; HRMS: C_25_H_27_N_4_O_3_S [*M*+H]^+^ calcd 463.1804; found 463.1809.

#### 3-(2-Hydroxycyclohexyl)-6-(2-methoxy-6-((4-(1-methyl-1*H*-pyra-zol-4-yl)benzyl)oxy)phenyl)pyrimidin-4(3*H*)-one (26 c)

General procedure B. Purification by flash column chromatography (CH_2_Cl_2_ 100% → CH_2_Cl_2_/MeOH 8:2) yielded the titled product as a light yellow oil (22 mg, 35%). ^1^H NMR (CDCl_3_): *δ* = 8.16 (s, 1H), 7.67 (s, 1H), 7.51 (s, 1H), 7.39–7.31 (m, 2H), 7.24–7.19 (m, 3H), 6.64–6.55 (m, 2H), 6.50 (s, 1H), 5.02 (s, 2H), 4.48 (t, *J*=9.6 Hz, 1H), 3.86 (s, 3H), 3.83–3.75 (m, 1H), 3.73 (s, 3H), 3.32 (s, 1H), 2.06–1.94 (m, 2H), 1.84–1.59 (m, 3H), 1.44–1.20 (m, 3H); *m/z* MS (TOF ES^+^) 486.9 [*M*+H]^+^; LC-MS *t*_R_: 3.49; HRMS: C_28_H_31_N_4_O_4_ [*M*+H]^+^ calcd 487.2345; found 487.2347.

#### 6-(2-Hydroxy-6-((4-(1-methyl-1*H*-pyrazol-4-yl)benzyl)oxy)phenyl)-3-(2-hydroxycyclohexyl)pyrimidin-4(3*H*)-one (26 d)

General procedure B. Purification by flash column chromatography (CH_2_Cl_2_ 100% → CH_2_Cl_2_/MeOH 8:2), followed by preparative HPLC (eluent 5–100%) yielded the titled product as a colourless yellow oil (16 mg, 34%). ^1^H NMR (CDCl_3_): *δ* = 8.16 (s, 1H), 7.74 (s, 1H), 7.59 (s, 1H), 7.51 (s, 1H), 7.49–7.44 (m, 2H), 7.40–7.35 (m, 2H), 7.16 (t, *J*=8.3 Hz, 1H), 6.58 (dd, *J*=8.3, 0.9 Hz, 1H), 6.48 (dd, *J*=8.3, 0.8 Hz, 1H), 5.13 (s, 2H), 4.44 (br t, *J*=9.2 Hz, 1H), 3.92 (s, 3H), 3.89–3.82 (m, 1H), 2.25–2.15 (m, 1H), 2.05–1.95 (m, 1H), 1.88–1.68 (m, 3H), 1.50–1.33 (m, 3H); *m/z* MS (TOF ES^+^) 472.9 [*M*+H]^+^; LC-MS *t*_R_: 3.37; HRMS: C_27_H_29_N_4_O_4_ [*M*+H]^+^ calcd 473.2189; found 473.2167.

#### 6-(2-((6-(1*H*-Pyrazol-1-yl)pyridin-3-yl)methoxy)phenyl)-3-(2-hydroxycyclohexyl)pyrimidin-4(3*H*)-one (27 b)

General procedure B. Purification by flash column chromatography (CH_2_Cl_2_ 100% → CH_2_Cl_2_:MeOH 9:1) yielded the titled product as a colourless oil (276 mg, 89%). ^1^H NMR (CDCl_3_): *δ* = 8.47–8.45 (m, 1H), 8.29 (d, *J*=1.9 Hz, 1H), 8.13 (s, 1H), 7.87 (d, *J*=8.4 Hz, 1H), 7.76 (ddd, *J*=11.0, 8.1, 2.0 Hz, 2H), 7.67–7.64 (m, 1H), 7.30–7.24 (m, 1H), 7.00–6.95 (m, 2H), 6.91 (d, *J*=8.2 Hz, 1H), 6.38 (dd, *J*=2.5, 1.7 Hz, 1H), 5.03 (s, 2H), 4.45–4.33 (m, 1H), 3.98 (br s, 1H), 3.87–3.78 (m, 1H), 2.09–1.99 (m, 1H), 1.93–1.85 (m, 1H), 1.73–1.59 (m, 3H), 1.43–1.15 (m, 3H); *m/z* MS (TOF ES^+^) 443.9 [*M*+H]^+^; LC-MS *t*_R_: 3.22; HRMS: C_25_H_26_N_5_O_3_ [*M*+H]^+^ calcd 444.2036; found 444.2036.

#### 6-(2-((4-(1*H*-Pyrazol-1-yl)benzyl)oxy)phenyl)-3-(2-hydroxycyclo-hexyl)pyrimidin-4(3*H*)-one (27 c)

General procedure B. Purification by flash column chromatography (PET/EtOAC 8:2 → EtOAc 100%) yielded the titled product as a white solid (220 mg, 71%). ^1^H NMR ([D_6_]DMSO): *δ* = 8.56–8.49 (m, 2H), 8.00–7.95 (m, 1H), 7.87 (d, *J*=8.6 Hz, 2H), 7.76 (d, *J*=1.6 Hz, 1H), 7.62–7.56 (m, 2H), 7.47–7.42 (m, 1H), 7.27 (d, *J*=8.0 Hz, 1H), 7.13–7.07 (m, 1H), 7.03 (s, 1H), 6.59–6.52 (m, 1H), 5.29 (s, 2H), 4.95 (d, *J*=5.7 Hz, 1H), 4.28 (br s, 1H), 3.95 (br s, 1H), 2.05–1.97 (m, 1H), 1.84–1.65 (m, 4H), 1.38–1.24 (m, 3H); *m/z* MS (TOF ES^+^) 442.9 [*M*+H]^+^; LC-MS *t*_R_: 3.33; HRMS: C_26_H_27_N_4_O_3_ [*M*+H]^+^ calcd 443.2083; found 443.2079.

#### 4-(2-(Benzyloxy)phenyl)-1-(2-hydroxycyclohexyl)pyridin-2(1*H*)-one (27 d)

Synthesized as previously described in the literature.^[14]^

#### 3-(2-Methoxycyclohexyl)-6-(2-((4-(1-methyl-1*H*-pyrazol-4-yl) benzyl)oxy)phenyl)pyrimidin-4(3*H*)-one (28 a)

General procedure E. Purification by flash column chromatography (CH_2_Cl_2_ 100% → CH_2_Cl_2_/MeOH 9:1), followed by preparative HPLC (eluent 5–100%) yielded the titled product as a colourless oil (37 mg, 45%). ^1^H NMR (CDCl_3_): *δ* = 8.14 (s, 1H), 7.98 (dd, *J*=7.8, 1.8 Hz, 1H), 7.74 (d, *J*=0.6 Hz, 1H), 7.59 (s, 1H), 7.48–7.44 (m, 2H), 7.42–7.37 (m, 2H), 7.34 (ddd, *J*=8.3, 7.4, 1.8 Hz, 1H), 7.23 (d, *J*=0.6 Hz, 1H), 7.06 (td, *J*=7.6, 0.9 Hz, 1H), 7.04–7.00 (m, 1H), 5.19 (s, 2H), 4.39 (br s, 1H), 3.92 (s, 3H), 3.69 (br s, 1H), 3.25 (s, 3H), 2.37–2.29 (m, 1H), 2.07–1.99 (m, 1H), 1.95–1.73 (m, 3H), 1.49–1.24 (m, 3H); *m/z* MS (TOF ES^+^) 470.9 [*M*+ H]^+^; LC-MS *t*_R_: 3.44; HRMS: C_28_H_31_N_4_O_3_ [*M*+H]^+^ calcd 471.2396; found 471.2394.

#### 6-(2-((4-(1-Methyl-1*H*-pyrazol-4-yl)benzyl)oxy)phenyl)-3-(2-oxocy-clohexyl)pyrimidin-4(3*H*)-one (29 a)

General procedure F. Purification by flash column chromatography (CH_2_Cl_2_ 100% → CH_2_Cl_2_/MeOH 9:1), followed by preparative HPLC (eluent 5–100%) yielded the titled product as a colourless oil (33 mg, 41%). ^1^H NMR (CDCl_3_): *δ* = 8.06 (d, *J*=0.4 Hz, 1H), 8.00 (dd, *J*=7.8, 1.8 Hz, 1H), 7.75 (d, *J*=0.6 Hz, 1H), 7.59 (s, 1H), 7.51–7.44 (m, 2H), 7.42–7.32 (m, 3H), 7.27 (d, *J*=0.7 Hz, 1H), 7.07 (td, *J*=7.7, 0.9 Hz, 1H), 7.04–6.99 (m, 1H), 5.66 (dd, *J*=12.5, 5.9 Hz, 1H), 5.23–5.15 (m, 2H), 3.93 (s, 3H), 2.71–2.64 (m, 1H), 2.60–2.51 (m, 1H), 2.44–2.36 (m, 1H), 2.26–2.18 (m, 1H), 2.17–2.00 (m, 2H), 1.98–1.73 (m, 2H); *m/z* MS (TOF ES^+^) 454.9 [*M*+H]^+^; LC-MS *t*_R_: 3.38; HRMS: C_27_H_27_N_4_O_3_ [*M*+H]^+^ calcd 455.2083; found 455.2085.

### Pharmacology

#### Whole-cell radioligand binding assays

FlpIn Chinese hamster ovary (CHO) cells stably expressing the hM_1_ mAChR were grown in Dulbecco’s modified Eagle’s medium (DMEM) (Invitrogen, Carlsbad, CA) supplemented with 5% foetal bovine serum (FBS) (Thermo-Trace, Melbourne, Australia) and 300 μg/mL G418 (Invitrogen, Carlsbad, CA). The cells were plated at 50,000 cells per well of 96-well white clear bottom plates (Greiner Bio-one, Kremsmünster, Austria), and were grown overnight. The following day, cells were washed twice with Phosphate Buffered Saline (PBS), and incubated with increasing concentrations of ACh (Sigma) in the absence or presence of increasing concentrations of each modulator and 0.2 nM [^3^H]NMS (PerkinElmer Life Sciences) in binding buffer (20 mM HEPES, 100 mM NaCl, 10 mM MgCl_2_, pH 7.4) for 5 h at room temperature. Nonspecific binding was determined using atropine at the final concentration of 10 μM. The assays were terminated by rapid removal of the unbound radioligand followed by two washes with 100 μL/well ice-cold 0.9% NaCl buffer. Radioactivity was determined by addition of 100 μL/well Ultima gold (PerkinElmer Life Sciences) and counting in a MicroBeta plate reader (PerkinElmer Life Sciences).

#### *IP*_*1*_ accumulation assays

FlpIn CHO cells stably expressing the hM_1_ mAChR were seeded at 25000 per well of 96-well transparent cell culture plates and grown overnight. The following day, cells were pre-incubated with IP_1_ stimulation buffer (1 mM CaCl_2_, 0.5 mM MgCl_2_, 4.2 mM KCl, 146 mM NaCl, 5.5 mM D-Glucose, 10 mM HEPES and 50 mM LiCl, pH 7.4) for 1 h at 37°C. Cells were then stimulated with increasing concentration of ACh in the absence or presence of increasing concentrations of each modulator for 1 h at 37°C. The reactions were terminated by removal of the stimulation buffer and addition of 50 μL of lysis buffer (50 mM HEPES pH 7.0, 15 mM KF, 1.5% *v/v* Triton-X-100, 3% *v/v* FBS, 0.2% *w/v* BSA). Seven μL of cell lysates were transferred into wells of 384-well Proxiplates (Perki-nElmer Life Sciences), and IP_1_ levels were measured using the IP-One assay kit (Cisbio, Codolet, France). The lysates were incubated with 1.5 μL of the cryptate-labelled anti-IP_1_ antibody and 1.5 of the ■ ■d2 ‘*D*_*2*_’ *?*■ ■-labelled IP_1_ analogue for 1 h at 37°C. The emission signals were measured at 620 and 665 nm after excitation at 340 nm using an Envision multilabel plate reader (PerkinElmer Life Sciences). The signal was expressed as the HTRF ratio, interpolated from the standard curve, and normalized to the maximum response to ACh.

#### β-Arrestin 2 recruitment assays

Parental FlpIn CHO cells were plated at 30000 cells/well of 96-well white Culture plates (Perkin Elmer Life Sciences) and transiently transfected with 10 ng/well of M_1_ *Renilla luciferase* (Rluc)-8 and 40 ng/well of YFP-β-arrestin 2 using linear polyethyleneimine (PEI/DNA ratio 6:1) diluted in 150 mM NaCl. DNA:PEI complexes were formed by 15 min incubation at room temperature then added to the cells and incubated at 37°C for 24 h prior to use. Cells were then washed and equilibrated in Hanks’ balanced salt solution for 1 h at 37°C. Coelenterazine h at a final concentration of 5 μM was added to each well, followed by addition of increasing concentrations of ACh in the absence or presence of increasing concentrations of each modulator. Lumines-cence and fluorescence signals were measured 5 min after agonist stimulation using the LUMIstar Omega plate reader (BMG LabTech, Offenburg, Germany). Light emission was detected at 475±30 nm for Rluc8 and 535±30 nm for YFP and BRET signal was calculated as the ratio of the light emitted by YFP to the light emitted by Rluc8. Data were then normalized to the maximum response to ACh.

#### Data analysis

All data were analysed by using Prism 7 (GraphPad Software, San Diego, CA).

Initial assessment of M1 PAM activity was analysed using the 3-parameter logistic equation to quantify baseline levels and potency estimates (pEC_50_) of ACh in absence or presence of 1 and 10 μM of PAM. Changes in both baseline and pEC_50_ were quantified by subtracting the estimates at each M1 PAM concentrations with the control values. Propagation of the error on baseline and potency values were determined as follows [Eq. (1)]:

$$\sigma_{\text{Δ}}=\sqrt{\sigma_{PAM}^{2}+\sigma_{cont.\;}^{2}}$$

where *σ*_Δ_ is the standard error of the mean (SEM) for the change in value, and *σ*_PAM_ and *σ*_cont._ are the SEM from the three-parameter logistic equation analysis in presence or absence of M1 PAM, respectively.

Binding interaction studies with allosteric ligands were fitted to the following allosteric ternary complex model [Eq. (2)]^[15]^

$$Y=\frac{{\text{B}}_{\max}|\text{A}|}{[\text{Λ}]+\left[\frac{{\text{K}}_{\text{A}}{\text{K}}_{\text{B}}}{\alpha^{\prime }[\text{B}]+{\text{K}}_{\text{B}}}\right]\left[1+\frac{\left[\text{I}\right]}{{\text{K}}_{1}}+\frac{\left[\text{B}\right]}{{\text{K}}_{\text{B}}}+\frac{\alpha [\text{I}][\text{B}]}{{\text{K}}_{\text{I}}{\text{K}}_{\text{B}}}\right]}$$

where *B*_max_ is the total number of receptors, [A], [B] and [I] denote the concentrations of radioligand, allosteric modulator, and orthosteric ligand, respectively; *K*_A_, *K*_B_ and *K*_I_ are their respective equilibrium dissociation constants. *α*’ and *α* are the affinity cooperativity factors between the allosteric ligand and radioligand or the allosteric modulator and ACh, respectively. Values of *α* or *α*’ > 1 denote positive cooperativity, values < 1 but > 0 denote negative cooperativity, and a value of 1 indicates neutral coopera-tivity.

Functional interaction studies between ACh and allosteric modulators in IP_1_ and β-arrestin 2 recruitment assays were analysed according to a three-parameter logistic equation or the following operational model of allosterism and agonism [Eq. (3)].^[16]^

$$E=\;\text{Basal}\;+\frac{\left({\text{E}}_{\text{m}}-\;\text{Basal}\;\right){\left([\text{A}]\left({\text{K}}_{\text{B}}+\alpha \beta [\text{B}]\right)+\tau_{\text{B}}[\text{B}]{\text{EC}}_{50}\right)}^{\text{n}}}{{\text{EC}}_{50}\text{n}{\left({\text{K}}_{\text{B}}+[\text{B}]\right)}^{\text{n}}+{\left([\text{A}]\left({\text{K}}_{\text{B}}+\alpha \beta [\text{B}]\right)+\tau_{\text{B}}[\text{B}]{\text{EC}}_{50}\right)}^{\text{n}}}$$

where *E*_m_ is the maximal possible system response, and Basal is the response in the absence of agonist. [A] and [B] are concentrations of orthosteric and allosteric ligands, respectively. *K*_B_ is the equilibrium dissociation constant of allosteric ligand, and EC_50_ is the concentration of orthosteric agonist required to achieve half maximal response. *α* and *β* represent the magnitude of the allosteric effects on orthosteric ligand affinity and efficacy, respectively; *τ*_B_ is the efficacy of allosteric ligand, and *n* is the slope factor of the transducer function that links occupancy to response. The application of this simplified equation is only valid if the orthosteric agonist is a full agonist both in the absence and presence of all concentrations of modulator,^[16b]^ which was the case for this study.

All potency, affinity, efficacy, and cooperativity values were estimated as logarithms,^[17]^ and statistical differences were determined using two-tailed *t*-test with Holm-Sidak post-hoc or one-way analysis of variance with Dunnett’s multiple comparison post-hoc test, where appropriate. A value of p< 0.05 was considered statistically significant.

#### In vivo exposure studies in mice

The study was performed using 10–12 weeks old male C57Bl/6 J naïve mice in accordance with the Australian Code of Practice for the Care and Use of Animals for Scientific Purposes, with procedures approved by the Animal Ethics Committee of the Monash Institute of Pharmaceutical Sciences. The compounds (**2, 7 f** or **13c**, dosing at 10 mg/kg; BQCA, dosing at 20 mg/kg–the same dose used in our previous animal behavioural study^[18]^ were dissolved in 10% DMSO, 1.1% Tween 80 and 21.8 mM Tris buffer, and administrated in mice by the intraperitoneal (IP) route at the volume of 0.1 mL/10 g. Mice were euthanised at either 20 or 90 min (45 or 90 min for BQCA) post dosing by cardiac puncture and cervical dislocation under gaseous anaesthesia (*n* = 3 /drug/time point). The concentration of the testing compounds in plasma and brain homogenate were determined using ultra-performance liquid chromatography/mass spectrometry (LC/MS). The concentration of the M1 PAMs, that is, BQCA, **2, 7 f** and **13c** in the brain parenchyma were corrected by a subtraction of the compound within the brain vasculature as detailed in our previous study.^[18]^ The *K*_p_ was then calculated using the formula: *K*_p_ =concentration in the brain [μM]/concentration in the plasma (μM), assuming brain density of 1 g/mL. The unbound fraction was determined using rapid equilibrium dialysis. Mouse (C57Bl/6 J) plasma and brain homogenate was spiked with BQCA or compound **2** and dialysed for 6 h against PBS. Concentrations in the dialysate and donor samples at the end of the dialysis period were determined using LC/MS. The *K*_puu_ value was then calculated using the formula: *K*_puu_ = *K*_p_ x [unbound fraction (brain)/unbound fraction (plasma)].

## Figures and Tables

**Table 1:** Pharmacological evaluation of 4-phenylpyridin-2-one analogues with modification to the central core.

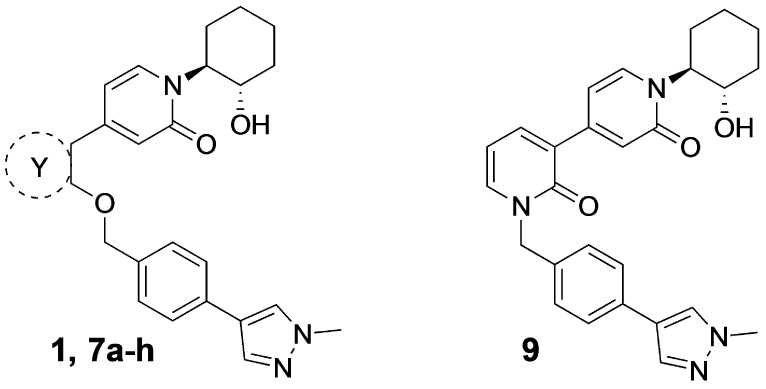					
Cpd	Y	ΔpEC_50_ [1 μM]^a^	Δbaseline [1 μM]^b^	ΔpEC_50_ [10 μM]^a^	Δbaseline [10 μM]^b^
1	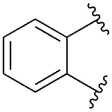	0.88±0.09	15.8±2.3	1.31±0.15	58.3±2.3
7a	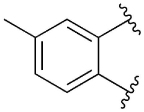	– 0.17±0.07*	0.47±2.0*	0.41±0.07*	4.4±2.0*
7b	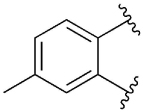	– 0.11±0.07*	3.5±1.9*	0.75±0.08	10.7±2.0*
7c	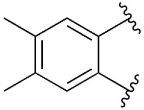	– 0.07±0.07*	–1.7±1.9*	0.41±0.08*	2.8±1.9*
7d	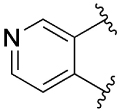	0.04±0.09*	–1.09±2.5*	0.21±0.10*	1.24±3.0*
7e	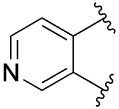	1.17±0.22	41.9±4.4*	1.47±0.35	79.4±2.9*
7f	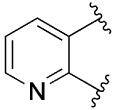	0.76±0.07	4.8±1.9*	1.41±0.10	20.5±2.5*
7 g	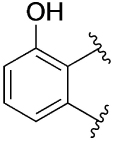	0.14±0.19*	12.8±4.4	0.35±0.34*	45.8±4.8*
7 h	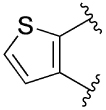	1.88±0.37*	70.8±4.0*	2.28±0.77*	90.3±3.1*
9		0.12±0.09*	5.0±2.0*	0.69±0.08	5.6±2.0*

Data represent the mean ± SEM of 3 independent experiments performed in duplicate. Changes in baseline and potency were calculated by substracting the values in absence of M_1_ PAM to the one in presence of either 1 or 10 μM. Propagation of the error on each values was calculated accroding to Equation (1) in the Experimental Section.

[a] Change in the negative logarism of ACh (pEC_50_) in presence of M_1_ PAM compared to control curve.

[b] Change in basal response in presence of M_1_ PAM compared to control curve, expressed as % of ACh maximal (*E*_max_) response.

* Significantly different (*p*<0.05) from the corresponding values for lead compound **1**, one-way ANOVA with Dunnett’s post-hoc test.

**Table 2:** Pharmacological evaluation of 6-phenylpyrimidin-4-one analogues with modification to the central core.

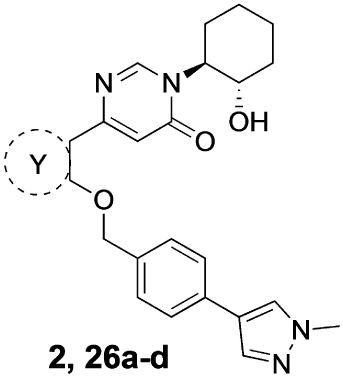					
Cpd	Y	ΔpEC_50_ [1 μM]^a^	Δbaseline [1 μM]^b^	ΔpEC_50_ [10 μM]^a^	Δbaseline [10 μM]^b^
2	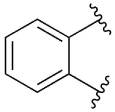	1.52±0.14	55.1±2.4	1.28±0.42	85.3±2.4
26a	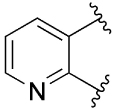	ND	100	ND	100
26b	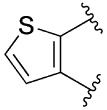	ND	100	ND	100
26c	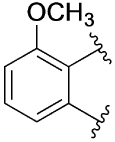	0.02±0.06*	1.37±1.6*	– 0.09±0.06*	5.99±1.7*
26d	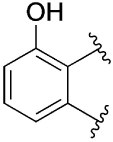	1.49±0.42	82.1±3.0*	ND	100

Data represent the mean ± SEM of 3 independent experiments performed in duplicate. Changes in baseline and potency were calculated by substracting the values in absence of M_1_ PAM to the one in presence of either 1 or 10 μM. Propagation of the error on each values was calculated accroding to Equation (1) in the Experimental Section.

[a] Change in the negative logarithm of ACh (pEC_50_) in presence of M_1_ PAM compared to control curve.

[b] Change in basal response in presence of M1 PAM compared to control curve, expressed as % of ACh maximal (*E*_max_) response. ND: not determined due to high agonist activity of the modulator, reaching 100% ACh maximal response (*E*_max_).

* Significantly different (*p* < 0.05) from the corresponding values for lead compound **2**, one-way ANOVA with Dunnett’s post-hoc test.

**Table 3:** Pharmacological evaluation of analogues with modification to the top motif.

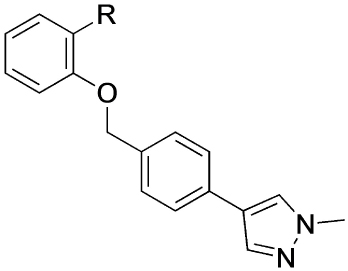					
Cpd	R	ΔpEC_50_ [1 μM]^a^	Δbaseline [1 μM]^b^	ΔpEC_50_ [10 μM]^a^	Δbaseline [10 μM]^b^
1	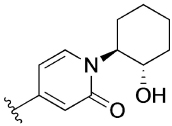	0.88±0.09	15.8±2.3	1.31±0.15	58.3±2.3
13b	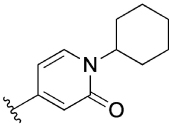	– 0.11±0.06*	5.0±1.7*	0.17±0.06*	–1.9±1.7*
13c	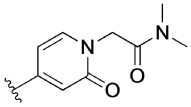	0.73±0.08	2.1±2.1*	1.33±0.17	63.6±2.3
13d	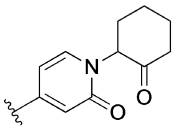	0.29±0.05*	4.8±1.4*	1.31±0.06	27.0±1.5*
14	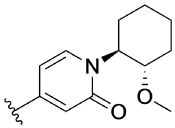	0.03±0.06*	5.8±1.5*	0.96±0.07	22.1±1.6*
19	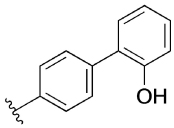	– 0.08±0.06*	– 4.8±1.7*	0.27±0.06*	– 0.6±1.7*
2	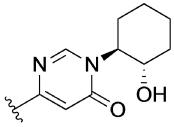	1.52±0.14	55.1±2.4	1.28±0.42	85.3±2.4
28a	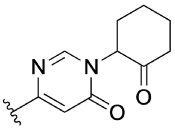	0.40±0.24^‡^	53.7±3.8	0.26±0.55^‡^	72.9±3.6^‡^
29a	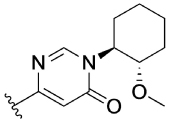	0.37±0.59^‡^	72.1±4.4^‡^	ND	ND

Data represent the mean ± SEM of 3 independent experiments performed in duplicate. Changes in baseline and potency were calculated by substracting the values in absence of M1 PAM to the one in presence of either 1 or 10 μM. Propagation of the error on each values was calculated accroding to Equation (1) in the Experimental Section.

[a] Change in the negative logarism of ACh (pEC_50_) in presence of M_1_ PAM compared to control curve.

[b] Change in basal response in presence of M_1_ PAM compared to control curve, expressed as% of ACh maximal (*E*_max_) response.

* Significantly different (*p* < 0.05) from the corresponding values for lead compound **1**, one-way ANOVA with Dunnett’s post-hoc test. ‡ Significantly different (*p* < 0.05) from the corresponding values for lead compound **2**, one-way ANOVA with Dunnett’s post-hoc test.

**Table 4:** Pharmacological evaluation of 6-phenylpyrimidin-4-one analogues with modification to the top and pendant moiety

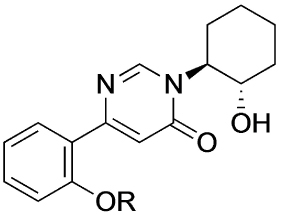					
Cpd	R^2^	ΔpEC_50_ [1 μM]^a^	Δbaseline [1 μM]^b^	ΔpEC_50_ [10 μM]^a^	Δbaseline [10 μM]^b^
2	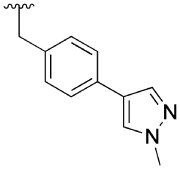	1.52±0.14	55.1±2.4	1.28±0.42	85.3±2.4
27b	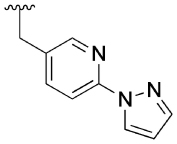	0.96±0.18	38.5±3.8*	1.50±0.32	78.6±2.8
27c	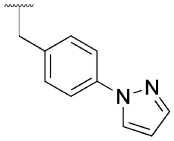	ND	100	ND	100
27d	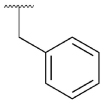	0.75±0.06	– 0.3±1.7*	1.83±0.11	54.6±1.9*

Data represent the mean ±SEM of 3 independent experiments performed in duplicate. Changes in baseline and potency were calculated by substracting the values in absence of M1 PAM to the one in presence of either 1 or 10 μM. Propagation of the error on each values was calculated accroding to Equation (1) in the Experimental Section.

[a] Change in the negative logarism of ACh (pEC_50_) in presence of M_1_ PAM compared to control curve.

[b] Change in basal response in presence of M1 PAM compared to control curve, expressed as % of ACh maximal (*E*_max_) response. ND, not determined due to high agonist activity of the modulator, reaching 100% ACh maximal response (*E*_max_).

* Significantly different (*p*<0.05) from the corresponding values for lead compound **2**, one-way ANOVA with Dunnett’s post-hoc test.

**Table 5:** Binding and functional allosteric parameters for selected PAMs at the M_1_ mAChR.

PAMs	3H]NMS binding		IP1 accumulation		β-arrestin 2 recruitment	
	p*K*_B_^[a]^ [*K*_B_ in μM]	log *α*_ACh_^[b]^ (*α*)	log *αβ*^[c]^ (*αβ*)	log *τ*_B_^[d]^ (τ_B_)	log *αβ*^ [c]^ (*αβ*)	log *τ*_B_^[d]^ (τ_B_)
BQCA	4.25±0.17 (56)	2.39±0.22 (245)	2.59±0.07 (389)	0.76±0.03 (6)	1.95±0.19 (89)	–0.03±0.10 (1)
1	4.96±0.12 (11)	2.05±0.17 (112)	2.00±0.10* (100)	0.60±0.03 (4)	1.81±0.09 (65)	–0.01±0.05 (1)
2	5.50±0.07* (3)	2.38±0.13 (240)	2.56±0.07 (363)	0.78±0.02 (6)	1.80±0.35 (63)	0.37±0.11 (2)
7f	4.42±0.39 (38)	2.38±0.39 (240)	2.50±0.08^#^ (316)	0.56±0.05 (4)	1.91±0.10 (81)	0.23±0.08 (2)
7h	5.22±0.10* (6)	2.58±0.18 (380)	2.75±0.11^#^ (562)	0.78±0.04 (6)	2.98±0.10* (955)	0.07±0.05 (1)
13c	4.78±0.22 (17)	1.86±0.28 (72)	2.07±0.08* (118)	0.45±0.03* (3)	1.74±0.11 (55)	–0.01±0.06 (1)
13d	4.53±0.15 (30)	1.72±0.20 (52)	1.48±0.11*^#^ (30)	–0.28±0.13*^#^ (1)	1.16±0.17* (14)	–0.61±0.32* (0.2)
26a	5.48±0.06* (3)	2.08±0.13 (120)	1.89±0.07*^ (28)	0.54±0.02^#^ (3)	1.98±0.10 (95)	0.28±0.04 (2)
27b	4.80±0.15 (16)	2.18±0.19 (151)	2.05±0.12*^ (112)	0.54±0.04^#^ (3)	1.75±0.11 (56)	–0.36±0.12^ (0.4)

Binding parameters were determined by using an allosteric ternary complex model [Eq (2)], and functional parameters using an operational model of allosterism and agonism [Eq (3)]. Data represent the mean ± SEM of at least three individual experiments in duplicate. In binding assays, logarithm of binding cooperativity between [^3^H]NMS and each modulator was fixed to -3 as the preferred model by F-test and in functional assays, the p*K*_B_ for each modulator was constrained to the values obtained in binding assays. There were no significant differences observed between log *α* and log *αβ* in IP_1_ or β-arrestin 2 assays, two-tailed *t*-test with Holm-Sidak post-hoc test.

[a] Negative logarithm of the allosteric modulator equilibrium dissociation constant.

[b] Logarithm of binding cooperativity between ACh and each modulator.

[c] Logarithm of functional cooperativity between ACh and each modulator.

[d] Logarithm of intrinsic efficacy of the modulator. * Significantly different from the corresponding values for BQCA as the reference PAM, one-way ANOVA with Dunnett’s post-hoc test.

# Significantly different from the corresponding values for lead compound **1**, one-way ANOVA with Dunnett’s post-hoc test. ^ Significantly different from the corresponding values for lead compound **2**, one-way ANOVA with Dunnett’s post-hoc test.

**Table 6:** Brain and plasma exposure of novel M_1_ mAChR PAMs in mice after IP administration.

Cpd (dose)	20 or 45 min post-dose		90 min post-dose		*K*_p _^[b]^
	*c*_brain_ [μM]^[a]^	*c*_plasma_ [μM]^[a]^	*c*_brain_ [μM]^[a]^	*c*_plasma_ [μM]^[a]^	
BQCA (20 mg/kg)	4.3–5.0 (0.7–0.9)	13.9–22.5 (0.7–1.2)	1.3–3.7 (0.2–0.6)	6.1–17.4 (0.3–0.9)	0.2–0.3 (0.6–1.0)
**2** (10 mg/kg)	0.7–1.2 (0.0)	15.0–16.5 (0.1)	0.0–0.1 (0.0)	0.7–1.1 (0.0)	0.0–0.1 (0.0–0.1)
**7f** (10 mg/kg)	0.3–0.4 (n.a.)	31.8–40.7 (n.a.)	0.1 (n.a.)	11.1–13.7 (n.a.)	0.0 (n.a.)
**13c** (10 mg/kg)	n.q.	25.3–46.5 (n.a.)	n.q.	4.9–5.4 (n.a.)	n.q.

[a] Range of total concentrations (and unbound concentrations in parentheses, where determined) in individual mice (*n* = 3) at that sample time.

[b] Range of *K*_p_ values (and *K*_p,uu_ in parentheses, where determined) in individual mice across both sample times (*n* = 6). n.q. Not quantifiable in brain parenchyma, hence *K*p could not be calculated. n.a. Not assessed
